# Control of Intermolecular Interactions toward the Production of Free-Standing Interfacial Polydopamine Films

**DOI:** 10.1021/acsami.3c05236

**Published:** 2023-07-25

**Authors:** Jakub Szewczyk, Visnja Babacic, Adam Krysztofik, Olena Ivashchenko, Mikołaj Pochylski, Robert Pietrzak, Jacek Gapiński, Bartłomiej Graczykowski, Mikhael Bechelany, Emerson Coy

**Affiliations:** † NanoBioMedical Centre, Adam Mickiewicz University, Wszechnicy Piastowskiej 3, 61-614 Poznan, Poland; ‡ Institut Européen des Membranes, IEM, UMR 5635, Univ Montpellier, CNRS, ENSCM Place Eugène Bataillon, 34095 Montpellier Cedex 5, France; § Faculty of Chemistry, Adam Mickiewicz University, Uniwersytetu Poznańskiego 8, 61-614 Poznań, Poland; ∥ Faculty of Physics, Adam Mickiewicz University, Uniwersytetu Poznańskiego 2, 61-614 Poznań, Poland; ⊥ Gulf University for Science and Technology, GUST, 32093 Hawally, Kuwait

**Keywords:** dopamine, oxidation, self-assembly, air−water interface, Brillouin light scattering

## Abstract

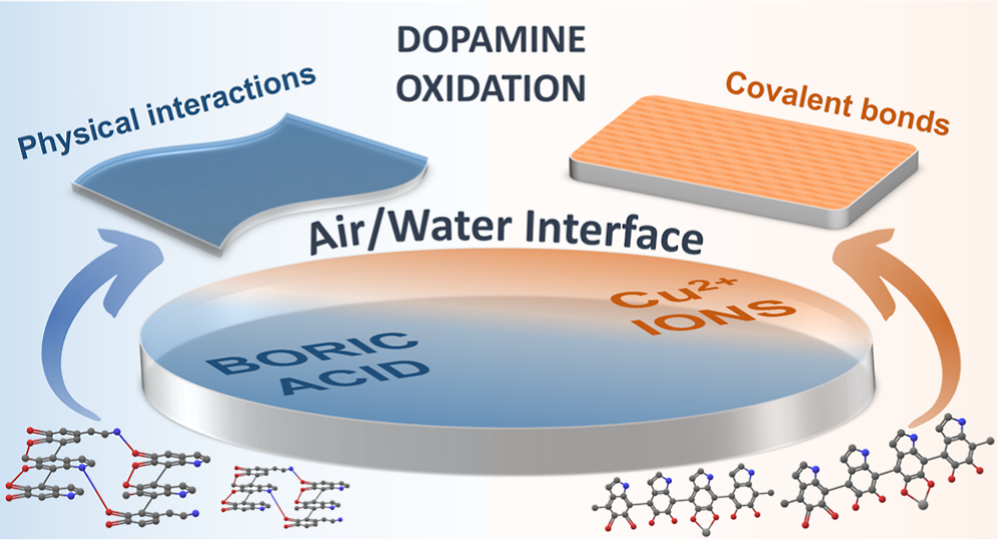

Aggregation of the polydopamine (PDA) molecular building blocks at the air/water interface leads to obtaining large surface nanometric-thin films. This mechanism follows two possible pathways, namely, covalent or non-covalent self-assembly, which result in a different degree of structure order and, consequently, different structural properties. Control of this mechanism could be vital for applications that require true self-support PDA free-standing films, for example, electrochemical sensing or membrane technology. Here, we are considering the impact of boric acid (BA) and Cu^2+^ ions on the mentioned mechanism exclusively for the free-standing films from the air/water interface. We have employed and refined our own spectroscopic reflectometry method to achieve an exceptionally high real-time control over the thickness growth. It turned out that BA and Cu^2+^ ions significantly impact the film growth process. Reduction of the nanoparticles size and their number was examined via UV–vis spectroscopy and transmission electron microscopy, showing a colossal reduction in the mean diameter of nanoparticles in the case of BA and a moderate reduction in the case of Cu^2+^. This modification is leading to significant enhancement of the process efficiency through moderation of the topological properties of the films, as revealed by atomic force microscopy. Next, applying infrared, Raman, and X-ray photoelectron spectroscopy, we presented small amounts of metal (B or Cu) in the final structure of PDA and simultaneously their vital role in the oxidation mechanism and cross-linking through covalent or non-covalent bonds. Therefore, we revealed the possibility of synthesizing films via the expected self-assembly mechanism which has hitherto been out of control. Moreover, modification of mechanical properties toward exceptionally elastic films through the BA-assisted synthesis pathway was shown by achieving Young’s modulus value up to 24.1 ± 5.6 and 18.3 ± 6.4 GPa, using nanoindentation and Brillouin light scattering, respectively.

## 1 Introduction

Polydopamine (PDA) is a nature-inspired polymer that gained exceptional attention in chemistry due to its unique adhesive properties and multiple surface functionalization ability.^1^ A relatively simple synthesis can produce various forms of this material or its analogues,^2−4^ that is, directly deposited coatings,^5,6^ functional nanoparticles,^7−9^ or free-standing films from the air/water interface.^10−13^ The latter is particularly interesting due to the self-assembly of PDA macromolecules at the interface, leading to high-quality nanometric-thin polymer films that can be transferred to various functional substrates.^14^

When applied to the modification of functional materials, PDA exhibits exceptional properties, such as hydrophilicity, anti-fouling,^15^ the enhancement of photocatalytic performance,^16^ and the increase of electrocatalytic activity,^17^ among others. Moreover, PDA thin coating on the transition-metal oxide-based electrode surface was recently shown to decrease charge-transfer resistance at the electrode–phosphate-buffered saline solution interface.^18^ However, so far, surface modifications with PDA leading to the improvement of the abovementioned properties have been based on the in situ deposition of coatings by oxidizing dopamine (DA) directly on the surface of the targeted material, preventing the integration on sensitive substrates. Nevertheless, an attractive alternative methodology is the possibility of ex situ functionalization of surfaces by PDA transferring, especially those that can tackle large-area functionalization.

Electrochemically grown PDA films have significant applicability in many fields, including sensing and electronics.^17^ However, their main drawback resides in the relatively small lateral side of the film produced by this method. The size of these films is directly limited by the size of the electrode used to oxidize DA. Conversely, the self-assembly of DA by oxidation at the air/water interface does not suffer from this limitation since the main limiting aspect is the water surface exposed to the air, typically in the range of tens of centimeters. Our recent work determined the influence of synthesis conditions for these PDA films to be transferable to functional substrates,^19^ showing similar thickness and roughness control as in the electrochemical deposition of PDA films but with the added value of retaining the so-called 2D-like ordering of the films.^14,19^ Molecular dynamics simulations have provided some insights into the macromolecular self-assembly at the air/water interface. However, it has not yet been shown how to control this process.

There are two possible pathways of aggregation of PDA molecules: (i) non-covalent self-assembly of the subunits forming H-bond and π–π interaction,^20^ (ii) covalent oxidative polymerization of the monomer subunits,^21^ and these pathways are simultaneously co-contributing to PDA formation.^22^ For potential applications of free-standing PDA films, to have the possibility of tuning the degree of supramolecular order is crucial, for example, toward obtaining a polymer more conjugated with π–π interactions, for the construction of even more efficient heterojunctions for photocatalytic applications.^23^ In turn, the covalent organic frameworks (COFs)—highly ordered structures with covalently linked organic units that can be topologically grown into various architectures—stormed the world of chemistry and functional materials in recent years.^24−26^ Moreover, it has already been proven that PDA can be obtained as COF, but only for nanoparticles, not for functional free-standing films.^27^ Therefore, we resolved to find chemical agents that would allow us to direct the synthesis toward covalent or non-covalent intramolecular interactions.

Boric acid (BA) was very recently successfully used to tune the diameter of PDA nanoparticles.^28^ Here, we are considering what impact BA can have on free-standing films from the air/water interface. Interestingly, at pH = 8.0, adding BA leads to reversible catechol-boron monocomplex formation, significantly increasing the share of non-covalent cross-linking of the structure.^29^ In turn, metallic ions (Na^+^, Ca^2+^, Mg^2+^, and Co^2+^) were used to modify free-standing PDA films, which greatly impacted the growth dynamics, interaction with nanoparticles suspended in the solution, and particulate properties such as hydrophilicity.^30^ However, in that study authors did not verify if metal ions (in particular Cu^2+^) could direct the DA oxidation process toward the desired intramolecular ordering type. Indeed, it was previously shown that Cu^2+^ might influence the structure of the colloids obtained in DA oxidation, reducing the degree of their self-organization.^31^

In this article, we analyze the impact of these two promising agents—BA and Cu^2+^ ions—on the synthesis and the resulting structure of free-standing PDA films from the air/water interface. The influence on thickness growth dynamics and mechanical properties of PDA films obtained at the air/water interface using BA and Cu^2+^ was further investigated. More importantly, we discovered that two divergent oxidation pathways lead to more favorable covalent or physical self-assembly of the molecules in the resulting material. Especially, the later ones are leading to exceptionally elastic films, with tuneable mechanical response. As mentioned above, the ability to precisely influence the structure of this material is crucial from the point of view of its application in photocatalysis, photovoltaics,^32,33^ and other emerging fields.

## 2 Materials and Methods

### 2.1 Chemical Reagents

Materials in all synthesis procedures were used without any further purifications. DA hydrochloride (CAS: 62-31-7, s, >98%), trizma base (CAS: 77-86-1, s, >99%), hydrochloric acid (CAS: 7647-01-0, l, 25%), copper(II) sulfate pentahydrate (CAS: 7758-99-8, s, ≥98.0%), acetic acid (CAS: 64-19-7, l, ≥99%), sodium acetate (CAS: 127-09-3, s, ≥99%), BA (CAS: 10043-35-3, s, ≥99.5%), sodium hydroxide (CAS: 1310-73-2, s, ≥98%), and silicon wafer (Si 100, CAS: 7440-21-3, s) from Sigma-Aldrich and ultrapure deionized water from a Hydrolab Ultra UV system were used.

### 2.2 Synthesis of the PDA Free-Standing Films

The film synthesis was carried out based on the optimized conditions determined in our previous work.^19^ DA (0.5 mg mL^–1^) in the form of DA hydrochloride powder was added to a Petri dish (7.5 cm in diameter, 2 cm in height) containing Tris buffer (10 mmol, 45 mL, pH = 8.0) or sodium acetate buffer (10 mmol, 45 mL, pH = 4.5) and an oxidation agent (BA or CuSO_4_, solid) in three different concentrations to achieve molar ratios of the DA/agent equal 1:1, 1:3, and 1:6. This is corresponding to 0.52, 1.56, and 3.12 mg mL^–1^ for copper(II) sulfate pentahydrate and 0.20, 0.60, and 1.20 mg mL^–1^ for BA, respectively. For clarity, all mixtures are summarized in Table 1. Stirring (300 rpm) occurred on a magnetic plate throughout the synthesis time (72 h), and a glass lid covered the vessel with a small gap to allow air exchange. The use of a buffer with an acidic pH of 4.5 in the case of Cu^2+^ as an oxidant is justified by the mechanism of DA oxidation in an acidic environment. Utilizing these ions in the presence of chloride ions and dissolved oxygen assumes an increase of the Cu(II)/Cu(I) redox potential.^34^ Note that it is not possible to use Cu^2+^ as an oxidant at basic pH = 8 because according to Pourbaix’s diagram of copper, the spontaneous formation of Cu(OH)_2_ occurs in that case.^35,36^ The optimized synthesis path (as mentioned above) allowed us to obtain homogeneous films with a size corresponding to the diameter of a Petri dish (7.5 cm). During the synthesis of all samples, an identical reaction was simultaneously carried out in the second vessel to collect the solution for UV–vis and dynamic light scattering (DLS) tests. After the desired oxidation time, the films were transferred in pieces to a silicon substrates (1 × 1 cm) using the simple scooping technique (Figure S1) that is a deposition of a fragment of the free-standing film on a substrate immersed in the solution using the gravity force. No additional washing was done, and purification of the samples of the so-transferred films was carried out. Although films obtained in the experiment are structurally PDA (not DA), for clarity, while describing all experimental results, we will be using the same nomenclature for mixtures and for films obtained from them.

**Table 1:** Composition of the Mixtures Used in the Experiment

name	buffer	pH	oxidation agent	DA/agent molar ratio
DA	tris	8.0	none	n.a.
DA/BA 1:1	tris	8.0, readjusted by NaOH	BA	1:1
DA/BA 1:3	tris	8.0, readjusted by NaOH	BA	1:3
DA/BA 1:6	tris	8.0, readjusted by NaOH	BA	1:6
DA/Cu 1:1	acetate	4.5	CuSO_4_ (Cu^2+^)	1:1
DA/Cu 1:3	acetate	4.5	CuSO_4_ (Cu^2+^)	1:3
DA/Cu 1:6	acetate	4.5	CuSO_4_ (Cu^2+^)	1:6

### 2.3 Physico-chemical Characterization

In our former publication,^19^ we described a home-made instrument for spectroscopic reflectometry (SR) built to study the dynamics of the free-standing film thickness growth at the air/water interface. Based on that experience, we have designed an improved and more compact version of such an instrument using commercially available units and avoiding glass parts to reach the UV range necessary to reduce the lower membrane thickness limit of detection. It is composed of the deuter-halogen light source AvaLight-DHc (Avantes), AvaSpec-Mini2048CL spectrometer (Avantes), and an optical fiber capable of operating in the broad UV–vis spectrum (220–800 nm). Previously, it was possible to determine with high accuracy the thickness of films starting from 50 nm. Lowering the wavelength to 200 nm allowed us to reveal the first maximum of the reflectance function already for 30 nm thick PDA film, as shown in Figure S1. For atomic force microscopy (AFM) measurements, we used an ICON-Bruker microscope with complementary Gwyddion software to analyze profilometry data, thickness, and roughness.^37^ To investigate the thickness of films after 72 h, we calculated the average of 10 measurements on randomly selected sections crossing the crack in the film with the exposed substrate. For roughness determination, we calculated the average of 10 measurements of the root-mean-square (rms) factor value in the area of 2 × 2 μm of the films. Transmission electron microscopy was performed with JEOL 1400 TEM. Samples were drop-cast on a copper grid (Lacey/Carbon film 200 mesh made by Ted Pella) directly from the reaction solution mixture and dried in a vacuum desiccator without any centrifugation. UV–vis measurements were performed using a LAMBDA 950 spectrophotometer (PerkinElmer). DLS and zeta-potential measurements were performed on the Zetasizer Nano ZS (Malvern Panalytical). The mean size of the nanoparticle hydrodynamic diameter was calculated basing on the number-based distribution. Due to the low DA concentration used in the experiment (0.5 mg mL^–1^), the samples for UV–vis and DLS measurements were not further diluted. The photograph of the remaining nanoparticle aggregates at the bottom and walls of the Petri dish was taken with a digital Xiaomi 50MP AI Quad Camera without additional intervention, after all liquid was slowly removed from the vial with a syringe. Raman spectroscopy was carried out employing a Renishaw instrument equipped with microscope enclosure RE04, 532 nm laser source, and Leica N PLAN 50×/0.5 objective lens. Exposure time was set to 0.1 s with 0.1% of the power of the laser source (corresponding to 20 μW), and the number of accumulations was 3. X-ray photoelectron spectroscopy (XPS) was performed in an ultra-high vacuum chamber (Specs) using a monochromatic X-ray source (Al anode). The vacuum in the analysis chamber was in 10^–9^ mbar range. The pass energy for the survey was set to 60 eV, while the high-resolution regions were collected for the pass energy of 20 eV. Data analysis was performed using the CasaXPS program. X-ray diffraction (XRD) characterization was executed with the use of an MRD-X’pert^3^ diffractometer (PANalytical), operating at 45 kV and 40 mA with a Cu Kα radiation source (wavelength of 1.54 Å). Nanoindentation of the film’s experiment was performed using a TI-950 (Hysitron) triboindenter with a Berkovich tip. Load and displacement curves were analyzed according to the Oliver and Pharr method.^38,39^ The methodology is described elsewhere.^40^ Finally, Brillouin light scattering (BLS) measurements were performed in the p–p backscattering geometry using the high-contrast tandem type Fabry–Perot interferometer (table stable) and the solid-state laser (Excelsior, Spectra-Physics, λ = 532 nm). The incident and backscattered light were focused and collected, respectively, using the microscope objective with a 10× magnification and a numerical aperture NA = 0.25. The incident power of the laser light was 0.8 mW. The spectrometer mirror spacing was set to 33 mm, and the scanning amplitude was 200 nm, allowing measurements in the ±1.7 GHz frequency range. All the spectra were recorded at a room temperature of 296.5 K (23.3 °C) and relative humidity of 39%. Ultra-thin films (below 30 nm) were needed for this experiment; therefore, the oxidation time was 12 h. When presenting data in the graphical form, error bars were included, except for the SR and UV–vis methods, where the error bars are smaller than the data point symbols.

## 3 Results and Discussion

**Figure 1:**
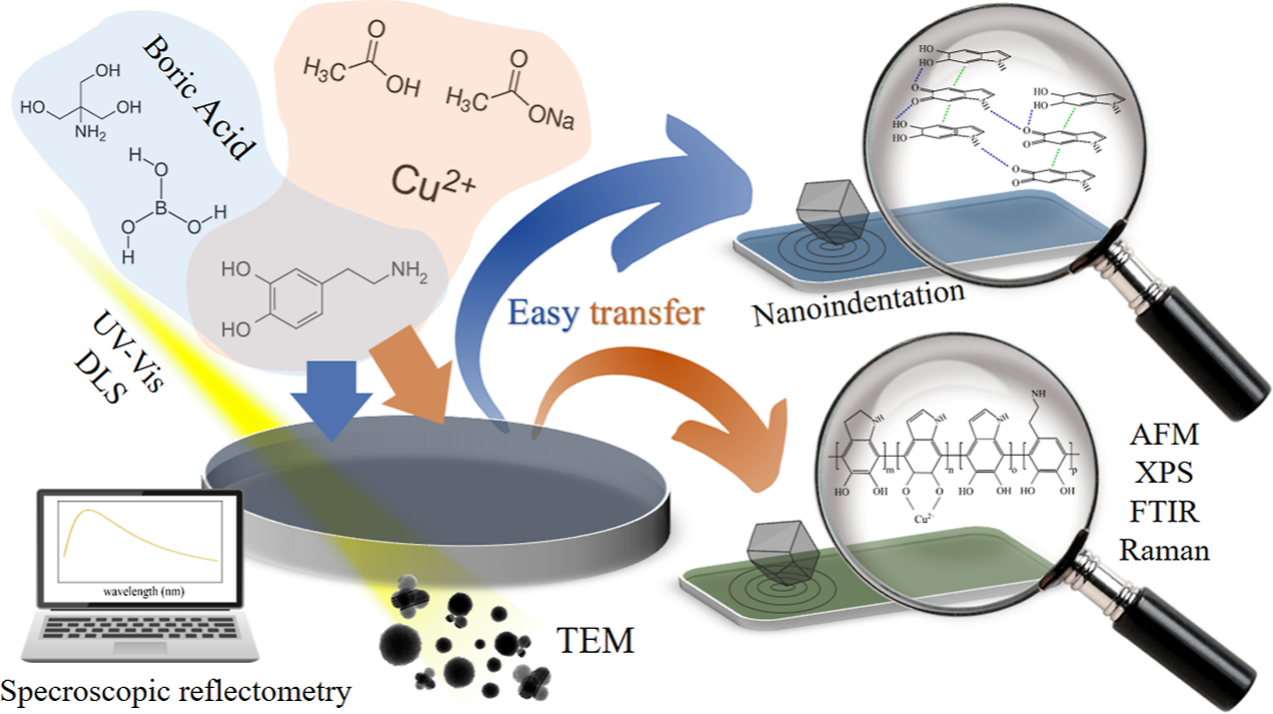
Scheme of the workflow—the synthesis proceeded under close observation of the oxidation, nanoparticles, and film growth dynamics, followed by a structural, chemical, and nanomechanical examination of the obtained films.

As illustrated in Figure 1, the work was divided into the following steps. We started with the production of DA, DA/BA, and DA/Cu free-standing films at the air/water interface. To follow the oxidation process, we measured the change in UV–vis absorbance of the solution and the growth dynamics of nanoparticles using the DLS and TEM methods. It was of a great importance because nanoparticle inclusions negatively affect the efficiency of the process and the quality of the PDA films at the air/water interfaces. At the same time, we studied the growth kinetics of films in situ at the interface using the non-destructive SR method. In the next step, we transferred the PDA thin films on silicon substrates to perform their topological (AFM) and structural and chemical (Raman, FTIR, and XPS spectroscopies) characterization. Moreover, we performed nanoindentation tests to check the flexibility and hardness of the obtained films. Finally, the elastic properties of the free-standing films were examined by BLS. Additionally, we present photographs of the films at the air/water interface and after transferring on the Si substrates (Figure S3).

**Figure 2:**
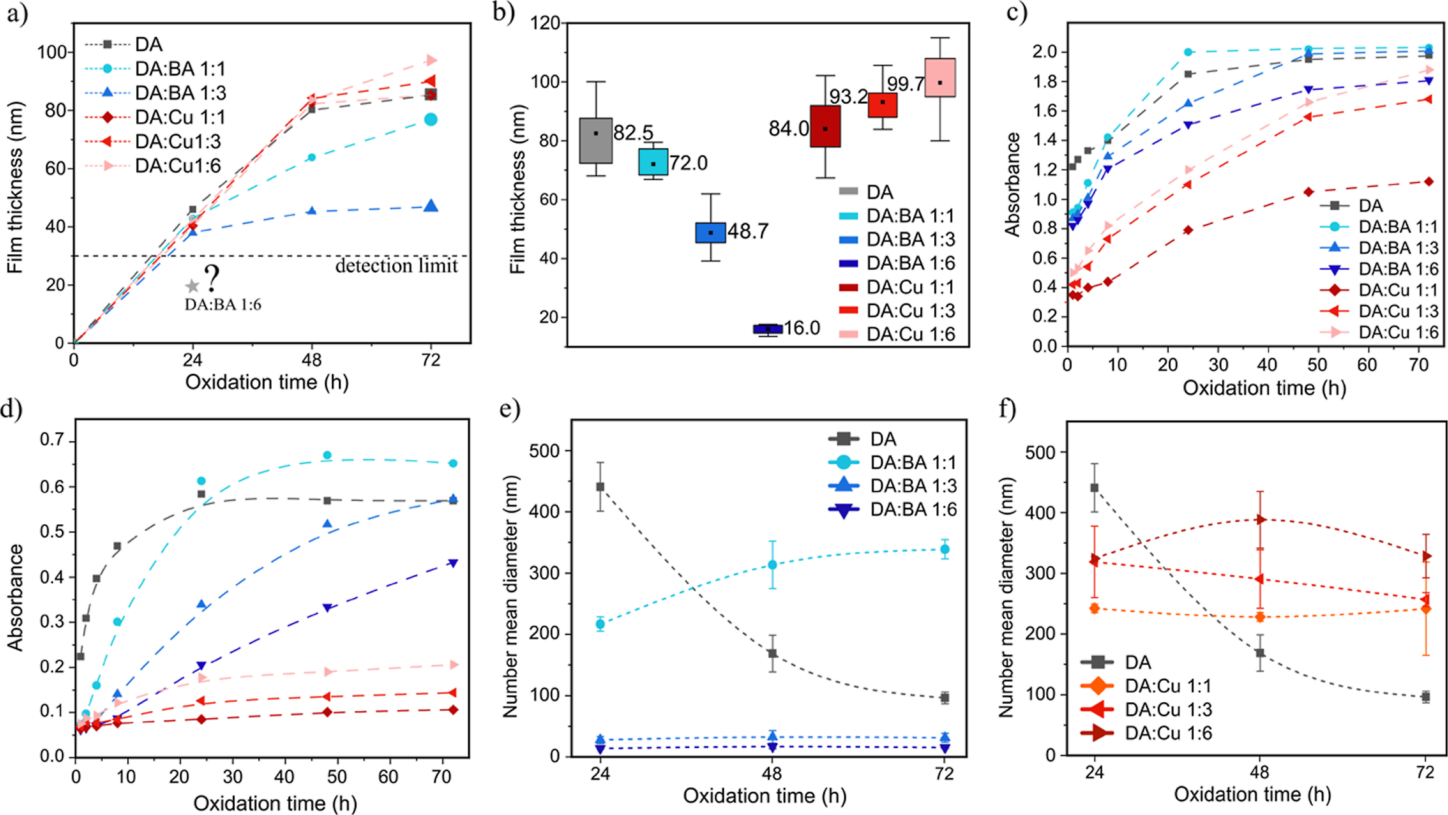
(a) In situ thickness growth of the films at the air/water interface measured by SR, (b) final thickness of the films transferred on Si substrates after 72 h measured by AFM, (c) UV–vis absorbance change during 72 h of the DA oxidation at the wavelength 305 and (d) 600 nm, and (e) DLS examination of the mean diameter of the PDA nanoparticles in the solution during 72 h of the DA oxidation modified with BA and (f) Cu^2+^. All dashed lines connecting data points on graphs are guide to the eye of the viewer only.

As mentioned above, we investigated the growth of the films in situ via SR (Figure 2a). In the first 24 h, the influence of BA and Cu^2+^ is not significant, except for DA/BA 1:6, where the growth of the film was almost completely inhibited. The last finding is in agreement with a recent observation that when the BA to DA ratio is higher than 3:1, the autoxidation of DA into PDA both in solution and at interfaces is terminated.^41^ In the next 24 h (24–48 h), in a mixture of DA and DA/Cu 1:1, 1:3, and 1:6, an almost linear increase in film thickness continues, while the growth rate of films DA/BA 1:3 and 1:6 slows down. Finally, in the last 24 h (48–72 h), we note a slow film thickness increase for all samples except for DA/Cu 1:6, where the growth rate is still significant. From our previous work, it follows that in the first step at the air/water interface, the forming DA tetramers quickly trap other planar molecules by physical forces (preferably π–π interactions), which induces quasi-ordered stacking of subsequently supplied molecules in the second step.^19^ For now, it can be concluded that Cu^2+^, as a good oxidant in these conditions, increases the efficiency of stacking the subsequent molecules to the already formed film at the air/water interface but does not significantly affect the efficiency of initial layer formation.

After 72 h, films from the air/water interface were transferred on a Si wafer and investigated via AFM for subsequent examination of the thickness (Figure 2b). The results are consistent with those obtained with SR. Using AFM, we managed to measure also the thickness of the DA/BA 1:6 film, which was not possible with SR due to the thickness detection limit (see Section 2.3). It turned out that an ultra-thin, continuous, easy-to-transfer film with a thickness of less than 20 nm was obtained in these conditions. It was completely transparent and colorless, almost invisible when transferred to silicon.

To study the DA oxidation and growth of the nanoparticles in the reaction solution, first, UV–vis spectra were measured in the range from 200 to 600 nm (Figure S4). The full spectrum of the DA oxidation mixture after 72 h shows broad absorption in this range because of the formation of the DA oxidation products that is 5,6-dihydroxyindole (DHI).^42^ The absorption at ∼350 nm corresponds to the quinone units in the PDA structure,^43,44^ providing evidence that a mixture of DHI and 5,6-indolequinone (IDQ) was obtained as assumed by the DA oxidation model. The absorption corresponding to boron-catechol bonds typically shows up at 490 nm, but here it is not visible due to the reversible boron-catechol interactions at pH = 8.0.^29^ Two wavelengths were chosen for plotting absorbance kinetics: 305 nm (Figure 2c) and 600 nm (Figure 2d). A strong peak at about 305 nm originates from the conversion of *o*-quinone to dopaminochrome,^45,46^ indicating an important intermediate oxidation step. Note that this step is so fast for DA and DA/BA samples that absorbance at *t* = 0 (corresponding to about 1 min) is significantly elevated, but this effect is limited for DA/Cu samples as the cyclization rate of *o*-quinone at acidic pH is slower.^34^ At 600 nm, only PDA nanoparticles are responsible for light absorption.^28,30^ At both wavelengths, DA alone with no agents shows fast absorbance change during the first 24 h and then plateau-like behavior. This suggests the fast and spontaneous generation of the PDA nanoparticles, consistent with the observed rapid color change of the solution. At λ = 305 nm, both the addition of BA and Cu^2+^ (except DA/Cu 1:1) resulted in a smoother curve and a similar final absorbance after 72 h, enabling to control the process. However, the very rapid increase in absorbance at *t* = 0 did not occur for Cu samples. This first step of the DA oxidation reaction may be partially coursed by aminochrome synthesis directly from active redox metal–DA adducts.^47^ Different mechanisms were reported for polyaniline film formation in the presence of Cu^2+^ ions, where deprotonated imine and amine nitrogen atoms form complexes with coordinating Cu^2+^ ions.^48^ This difference is due to the unique catechol-chemistry of DA, causing redox active metal ions to ligate to the adjacent hydroxyl groups of DA.^49^ BA (1:3 and 1:6) caused the λ = 600 nm curve to change shape and indicates the inhibition of the formation of nanoparticles, especially in the first 24 h. BA in a molar ratio 1:1 does not alter this behavior drastically. This is consistent with literature reports which indicate that for a drastic inhibition of DA oxidation to PDA, the required BA/DA ratio is higher than 3:1.^41^ Nevertheless, inhibition of the increase in absorbance at the wavelength 600 nm was achieved using Cu^2+^, which seems contrary to the assumptions as it should accelerate the oxidative generation of the PDA nanoparticles. To explain this feature, DLS measurements were performed (Figure 2e,f). The unexpected drop in the mean size of the nanoparticles in the case of pure DA after 24 h is caused by sedimentation of the large nanoparticle aggregates to the bottom and walls of the Petri dish (Figure S5). Aggregation of the PDA nanoparticles and increase of the stability of the nanoparticles/solution colloid can be achieved by positive ζ (zeta potential).^50^ Using Cu^2+^ as an oxidant in pH 4.5 leads to positively charged nanoparticles, as reported in the literature^31,51^ and confirmed in our study (Figure S6). Moreover, in the following sections, we will provide AFM and TEM evidence of the presence of large (>500 nm) nanoparticles in the pure DA reaction solution after 72 h. Back to the DLS results, in case of BA (Figure 2e), an appearance of 200 nm PDA aggregates and their slight growth within 72 h was observed for low concentration (1:1), but no such big aggregates of the nanoparticles throughout the whole oxidation time were noted for DA/BA 1:3 and DA/BA 1:6 mixtures. Based on the UV–vis and DLS results, it can be concluded that the increase in the diameter of the nanoparticles is stopped below 50 nm at the beginning of the oxidation. The slow further change in the color of the solution is caused only by the increase in the number of suspended nanoparticles. By analyzing graphs for Cu^2+^ containing samples (Figure 2f), we can see that for all mixture compositions, the growth of nanoparticles stopped at a level of a diameter below 200 for 1:1 and 400 nm for 1:3 and 1:6. This further confirms the weaker λ = 600 nm absorbance compared to pure DA that was observed in UV–vis tests. To conclude, based on the SR, UV–vis, and DLS results, we found that BA is a moderate inhibitor of the PDA free-standing film growth rate at the air/water interface and an exceptional inhibitor of the PDA nanoparticle size growth. Cu^2+^ ions in acidic pH (4.5) are slightly inhibiting the growth of the PDA nanoparticles but simultaneously accelerating the PDA free-standing film growth rate at the air/water interface.

**Figure 3:**
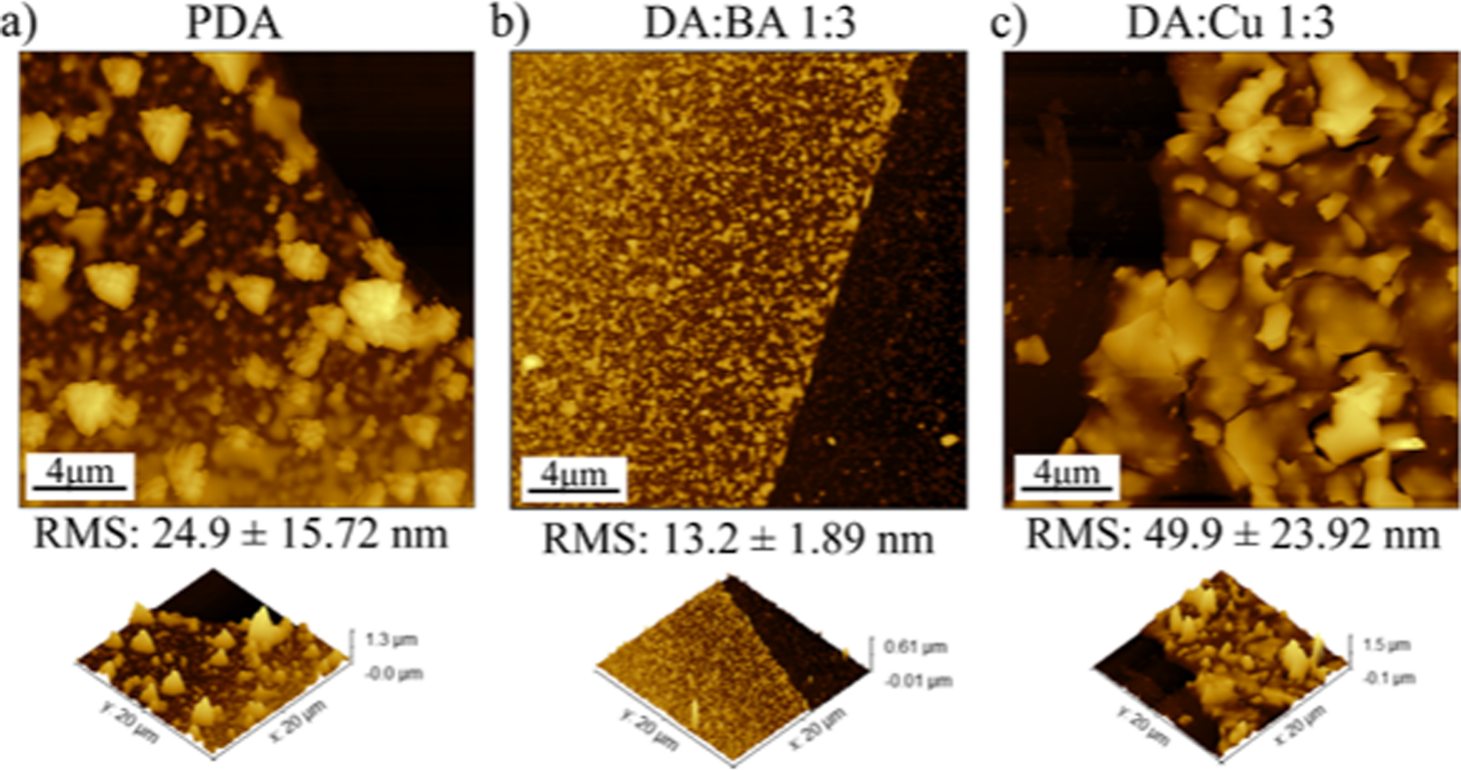
AFM topography images of the films after 72 h of oxidation at the air/water interface of the (a) PDA, (b) DA/BA 1:3, and (c) DA/Cu 1:3 solutions.

AFM was also applied to investigate the topography of the films (Figure 3). BA led to smooth and continuous film formation, showing an almost two times lower rms roughness coefficient than pure DA (Figure 3a,b). Although the nanoparticles in the DA/Cu 1:3 solution were smaller than that in pure DA, as demonstrated by UV–vis and DLS, these films’ roughness is the highest (Figure 3c). The reason is the scaly structure of the films. All films AFM topography images after transferring on the Si substrate (Figure S7) and corresponding roughness rms parameters (Table S1) are shown in Supporting Information.

**Figure 4:**
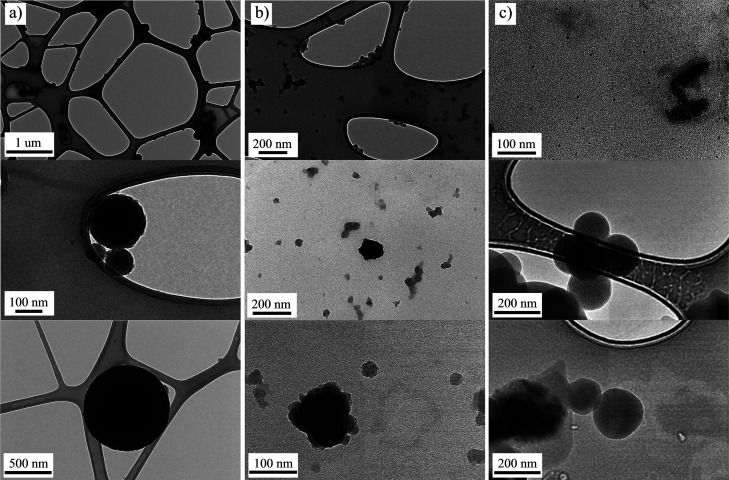
TEM images of the nanoparticles suspended in the (a) DA, (b) DA/BA 1:3, and (c) DA/Cu 1:3 solutions, after 72 h.

Transmission electron microscopy images of the nanoparticles formed in the solution and transferred dropwise on the TEM grids after 72 h of oxidation (Figure 4) confirm UV–vis and DLS findings. Note that no special protocol for obtaining and centrifuging nanoparticles was used. Therefore, all nanoparticles visible in the pictures were generated spontaneously. DA solution contains large single nanoparticles of a diameter >500 nm and numerous aggregates of the smaller nanoparticles (Figure 4a). In the DA/BA 1:3 mixture, there are very small nanoparticles (<50 nm), as shown before, but some nanoparticle aggregates can also be found, which, however, do not exceed 100 nm (Figure 4b). Electrostatically positively charged nanoparticles from the DA/Cu 1:3 mixture did not deposit so evenly on the TEM grids, but there were a lot of them on the grid rim. Therefore, the images are not perfectly sharp, but they still confirm previous observations, showing that the diameter of 200 nm dominates for the observed nanoparticles (Figure 4c).

**Figure 5:**
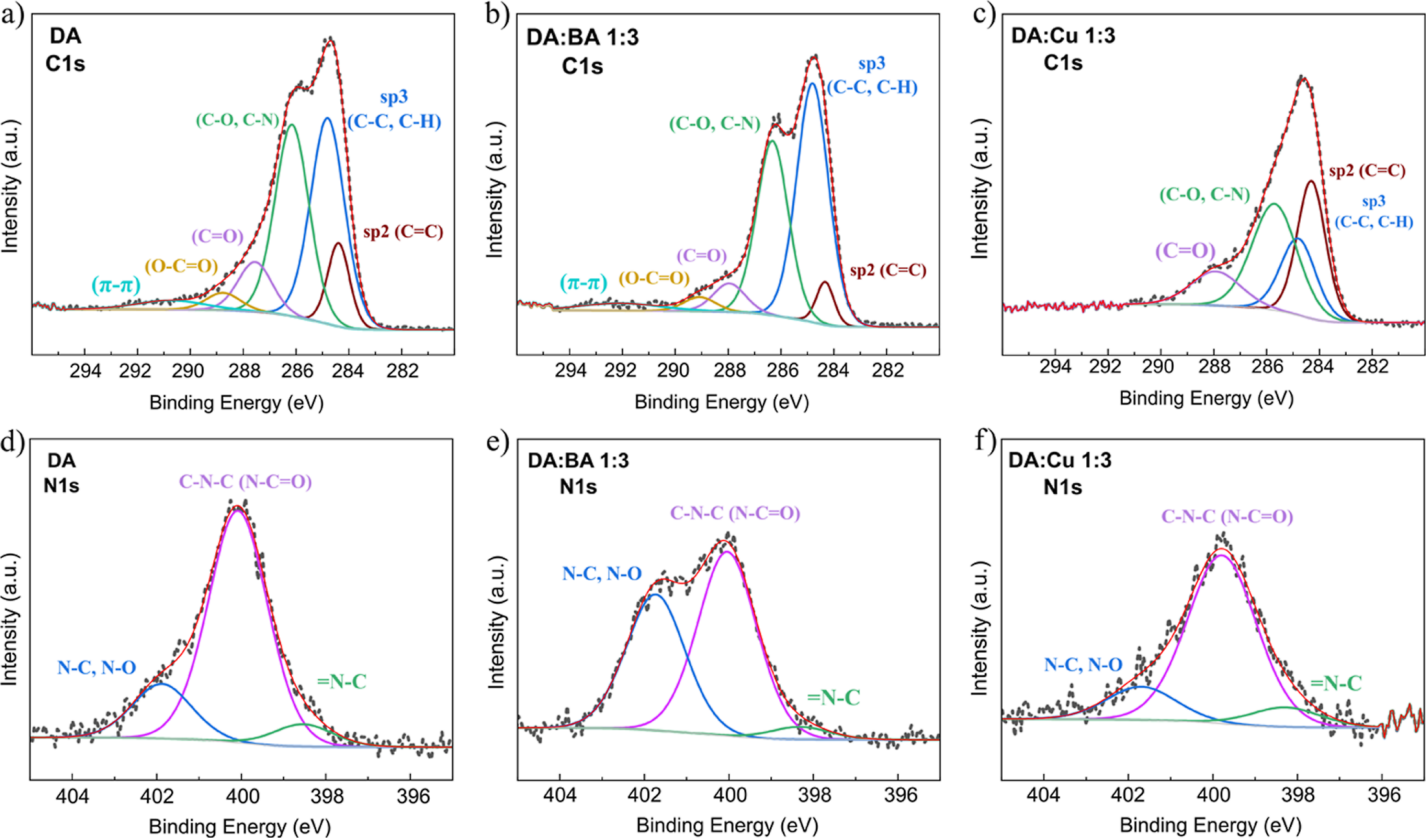
(a) X-ray photoelectron high-resolution spectra of the C 1s region for the DA, (b) DA/BA 1:3, and (c) DA/Cu 1:3 films, (d) high-resolution spectra of the N 1s region for the DA, (e) DA/BA 1:3, and (f) DA/Cu 1:3 films.

To investigate the influence of oxidation agents on the chemical structure of the PDA, we performed an XPS study (Tables S2–S4). The most important (C 1s and N 1s) spectra are shown in Figure 5a–c,d–f, respectively. Others (O 1s, Si 2p, Cl 2p, B 1s, Cu 2p, and S2p) can be seen in the Supporting Information (Figures S8 and S10). The first and most important observation is the presence of π–π bonds for DA and DA/BA 1:3 films, especially for the latter. This is an important indicator of the non-covalent self-assembly mechanisms of PDA aggregation through π–π and hydrogen bonds.^52^ Note that no π–π bonds presence was noted for DA/Cu 1:3, but on the other hand, a much larger contribution of sp^2^ carbon compared to sp^3^ carbon was detected. The occurrence of oxygen and hydrogen atoms bonded to carbon inside the graphene-like domains increases the C(sp^3^)/C(sp^2^) ratio.^53,54^ We have strong evidence that using BA promotes PDA aggregation through physical (non-covalent) self-assembly. This observation is supported by Table S5 summarizing sp^2^ and sp^3^ carbon XPS subpeaks areas, showing that sp^2^/sp^3^ ratio for DA/Cu 1:3 is almost 15 times higher than that for DA/BA 1:3. The PDA subunits form the hydrogen bonds and π–π interactions via the catechol groups and aromatic skeleton. In turn, strong oxidant Cu^2+^ promotes covalent oxidative polymerization of the monomer subunits, forming long chains with only rare connections through physical interactions. This is further confirmed by the analysis of N 1s high-resolution spectra of the films since the primary-amine part of the spectrum (N–C) is less significant for the DA/Cu 1:3 sample. A very recent study showed that primary amine contribution decreases with the oxidation time; thus, the decreased level indicates a higher oxidation level.^55^ Simultaneously, secondary amine abundance in the DA oxidation products indicates the elevated intramolecular cyclization rate.^56,57^ Therefore, the effect of the Cu^2+^ ions appears in both intermolecular and intramolecular interactions. Moreover, XPS showed that copper is present in the obtained films only in the form of the +2 ion (Figure S10). The structural units that make up the PDA can bind to Cu^2+^ via catechol groups.^49,58^ Apparently, catechol–Cu^2+^ complexes are not the basis of the structure of the obtained material because the EDX analysis showed a very small signal from copper (Figure S11). However, we believe that such complexes are present, but in low numbers, because the N 1s spectrum for DA/Cu 1:3 has an increased (=N–C) to (C–N–C) signal ratio in comparison to the other films (Figure 5d–f), and it was previously postulated that such effect could be due to the complexation of the catecholate group with a metal cation.^59^ Correspondingly, the presence of Cu^2+^ is confirmed also by the presence of a satellite (shake up) in the binding-energy range of 939–963 eV.^31^ No well-developed hybrid nanostructures resulting from the complexation process were formed, as shown in Figure 4c. This is due to weak metallic ion incorporation into the polymer structure. To change this behavior, a PBS buffer with a pH above 5 would be required.^60^ In turn, the boron-catechol mono-complexes trace amount in obtained DA/BA 1:3 film is credible on account of the mild XPS peak at around 192 eV, as previously reported.^28,61^ At this point, it is worth emphasizing that utilizing XPS we did not detect any signs of metallic boron or copper in the resulting material. Finally, our observations point out that the contribution of C–O and C–N bonds is almost identical for all films, which confirms the catechol and eumelanin chemistry of the obtained films.

**Figure 6:**
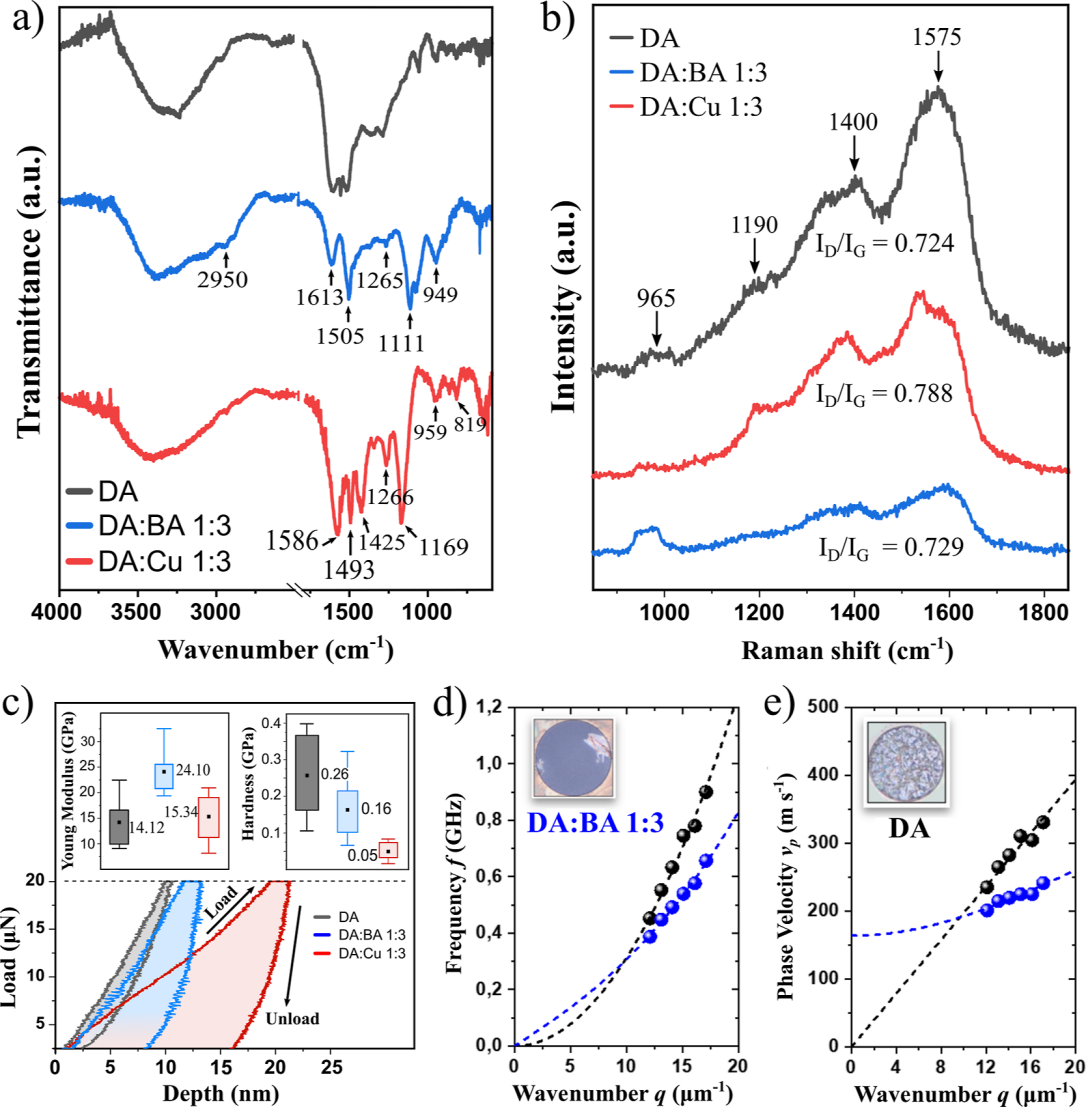
(a) Transmission Fourier-transform infrared spectra of the films, (b) Raman spectra of the films, (c) nanoindentation load–unload curves and box charts (inset) of the real Young modulus and hardness of the films, dispersion relations (d) *f*(*q*) and (e) *v*(*q*) of fundamental antisymmetric Lamb (flexural) waves measured with BLS spectroscopy for BA (blue points) and PDA (black points) membranes. The dashed lines represent the fit according to eqs 1 and 2. Inset images in (d,e) present optical microscopy images of the free-standing membranes DA/BA 1:3 and DA, respectively.

The FTIR spectra of our PDA films are shown in Figure 6a. The broad peak between 3200 and 3500 cm^–1^ indicates hydroxyl and N–H bonds in the catechol groups.^62^ However, this broad peak consists of individual bands,^63^ that is, “free” O–H stretch at ∼3525–3600 cm^–1^ and “bound” O–H stretch at 3360–3430 cm^–1^. For DA/Cu 1:3, the proportion of “free” O–H seems to be much higher, which can be seen in the deconvoluted part of the spectrum in the range 2800–3700 cm^–1^ (Figure S12). “Bonded” O–H is associated with the occurrence of hydrogen bonds. In turn, the presence of the 2950 cm^–1^ band is associated with CH_2_ stretching vibrations.^64^ Its low intensity for the DA/Cu 1:3 sample suggests weak H-bonding of the CH_2_ groups.^65^ In the case of DA/BA 1:3, it is a significant band, suggesting a large number of hydrogen bonds, while for DA, its intensity is moderate. Next, we can see a whole set of bands typical for PDA: 1613 cm^–1^ (shifted to 1586 cm^–1^ in the Cu sample) and 1505 cm^–1^ (shifted to 1493 cm^–1^ in Cu) corresponding to C=C resonance vibrations in the benzene rings^66^ and N–H vibration of the amine group,^64^ respectively. Importantly, C=C resonance vibrations are much stronger for DA/Cu 1:3 than that for DA/BA 1:3, while N–H vibration is similarly intensive. This confirms previous XPS observations. Moreover, 1111 and 1169 cm^–1^ bands originate from C–O stretching vibration.^64^ Finally, the peaks at 1425 and 1266 cm^–1^ are assigned to the phenolic C–O–H bending and stretching vibration, respectively.^67^ Additional peaks for DA/Cu 1:3 at lower frequencies, especially 819 cm^–1^, most probably originate from Cu–O stretching.^68,69^

Raman spectroscopy (Figure 6b) indicated the existence of two very important bands for all samples, namely, D band (1400 cm^–1^) attributed to structural defects in the hexagonal carbon lattice and G band (1575 cm^–1^) band linked with the in-plane vibration of the sp^2^ carbon atoms.^70^ The *I*_D_/*I*_G_ ratio was also calculated, showing less similarity to nanocrystalline graphite of DA/Cu samples, which suggests probably less planar-oriented structure than DA and DA/BA films.^14,71,72^ The peak at about 965 cm^–1^ indicates the C–H or O–H out-of-plane deformations. It is much less intense for DA/Cu, which confirms the lower impact of hydrogen bonds in the created structure. The band around 1190 cm^–1^ is assigned to the NH in-plane deformation mode originating from the pyrrole ring in the PDA structure and is the strongest for the DA/Cu 1:3 sample. Pyrrolic rings may participate in the formation of π–π or O–H interactions, which would weaken or shift the band. In the covalent structure, these rings are the basic elements of the construction of structural units (DHI, indolequinone). Moreover, the shape of so-called 2D (*G*′) peak was compared for DA and DA/Cu 1:3 films (Figure S13). DA/BA 1:3 was excluded from the analysis due to the low thickness of the films and thus significantly less intense bands in the whole spectrum. We can see that this peak is more intense for the DA sample, suggesting more planar interactions resulting from π–π bonding. Moreover, the peak for DA/Cu 1:3 is shifted toward lower wavenumbers, suggesting a higher oxidation level.^73^

X-ray diffractometry was performed to confirm the above information (Figure S14). As reported before, diffractograms for DA and DA/BA 1:3 show characteristic diffraction peaks for PDA free-standing films produced at the air/water interface.^14,19^ On the contrary, DA/Cu 1:3 diffractogram does not show any peaks which is in accordance with the lack of π–π interactions,^74^ as revealed by XPS.

To perform nanoindentation tests, we took advantage of the experience gained in our previous experiment.^14^ As our goal was to compare the effects of chemical environment (BA vs Cu^2+^) in the first place, we decided, as in previous chemical and structural studies, to compare samples prepared with identical molar ratios (1:3) and the same oxidation time (72 h). The film thicknesses were equal to 82.5, 48.7, and 93.2 nm for DA, DA/BA 1:3, and DA/Cu 1:3, respectively. The loading curves (Figure 6c) clearly indicate that the films deform in different ways. The DA film deforms the least, the DA/BA 1:3 film has a moderate deformation susceptibility, and the DA/Cu 1:3 film is almost plastic. The unloading curve can provide more information because we can extract sample Young’s modulus from its shape and slope. The highest Young’s modulus (24 GPa) was estimated for the film DA/BA 1:3. It is almost twice as high as the one obtained for DA (14 GPa); additionally, the load vs displacement curves show the high recovery of the DA film, with an almost ≈80% of elastic recovery, followed by DA/BA 1:3 with ≈40% and DA/Cu 1:3 with ≈25%. The shape of the curves also shows the higher plastic deformation on the DA/Cu 1:3 with respect of the other samples, despite the similar mechanical response. The average Young’s modulus for the DA/Cu 1:3 film (15 GPa) is slightly higher than that for the DA film, however within the measurement error (see the left inset in Figure 6c). In turn, the highest average hardness value was achieved for the DA film (0.26 GPa). The mean values for DA/BA 1:3 and DA/Cu 1:3 films were 0.16 and 0.05 GPa, respectively.

The elastic properties in the GHz frequency (*f*) range of BA and PDA membranes grown for 12 h were measured in a contactless and non-destructive manner by means of BLS spectroscopy probing thermally excited acoustic waves/phonons. By changing the scattering angle θ from 30 to 46°, the magnitude of the acoustic wavevector *q* was varied in the range of ∼12–17 μm^–1^ according to the formula *q* = 4π sin θ/λ, where λ = 532 nm is the wavelength of the probing laser light. Such obtained dispersion relations *f*(*q*) (Figure 6d) correspond to the antisymmetric, flexural Lamb waves (A0 mode). To determine the Young modulus *E* and the residual stress σ_0_, the experimental data were fitted with the eq 1(75)
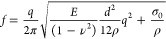
1where *v*_p_ = 2π*f*/*q*, is the phase velocity, *d* is the membrane thickness, ρ = 1750 kg m^–3^ is the film mass density, and ν = 0.25 stands for the Poisson ratio.^76^ For film thickness, we adopted the values estimated from AFM topography maps (Figures S15 and S16), *d*_BA_ = 10.5 ± 1.5 nm and *d*_PDA_ = 23 ± 3 nm for DA/BA and DA membranes, respectively. For the DA/BA 1:3 membrane, we obtained *E* = 18.3 ± 6.4 GPa and σ_0_ = 47 ± 6 MPa, while for the PDA membrane, *E* = 14.4 ± 4.0 GPa and σ_0_ = 0 as in this case, the data had not indicated any statistically significant residual stress. This last observation is better illustrated in the *v*_p_ vs *q* dependence since for *q* → 0, the eq 2 is
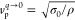
2(Figure 6e), and it is compatible with optical microscopy images shown in the insets in Figure 6d,e. The determined values of the Young modulus are in a reasonable agreement with the nanoindentation results. Unfortunately, the DA/Cu samples could not be measured by means of BLS because of their poor stability in free suspension. Membranes transferred on the substrates broke even when suspended over the smallest holes (1 μm), which confirms their poor elasticity, as observed in nanoindentation.

From this study, it can be concluded that the DA/BA 1:3 sample has competitive elastic properties, with a moderate elastic recovery, the highest Young’s modulus, and intermediate hardness. In turn, the DA/Cu 1:3 film does show low elastic recover, high plasticity, and low hardness. Noteworthy, it was shown that a more complex molecular structure promotes increasing hardness for the tested polymers,^77^ in agreement with the results of our experiment. Weakly physical cross-linked polymer materials more likely undergo a deformation induced by a reduction in molecular order under external stimuli,^78^ as observed for DA/Cu 1:3 films. More importantly, it was shown that the boron-catechol mono-complexes prevent oxidative covalent cross-linking and promote non-covalent cross-linking, resulting in a higher elasticity modulus of the obtained materials, which is confirmed in our experiment.

**Figure 7:**
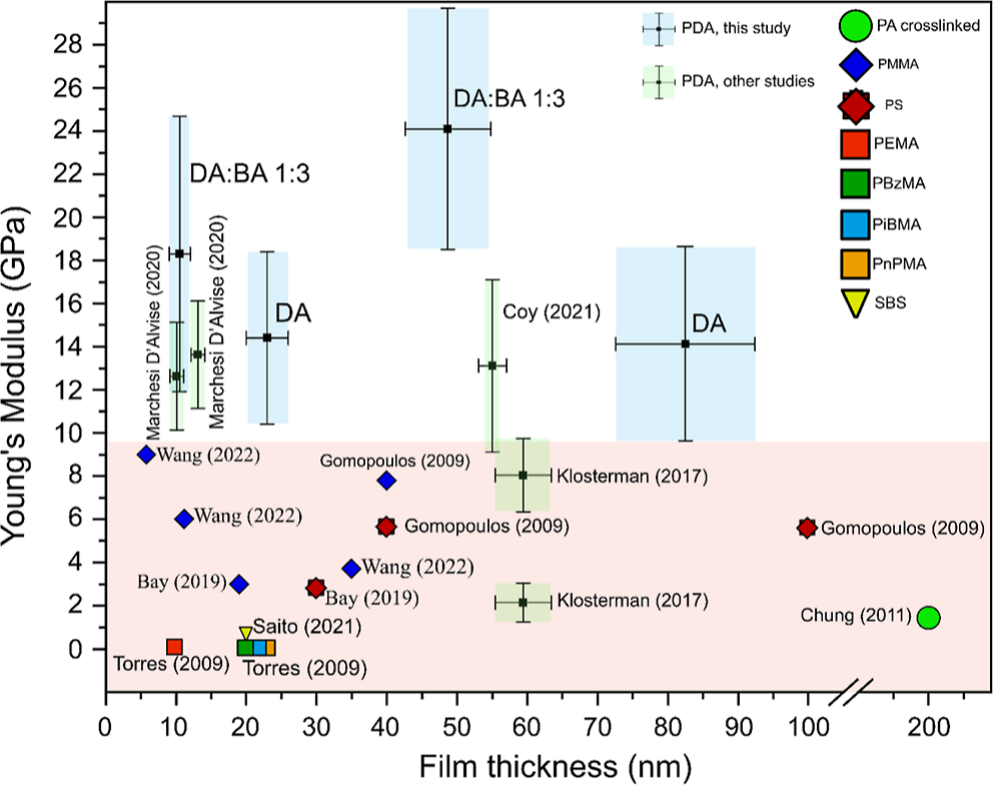
Comparison of Young’s modulus of selected thin (thickness less than 200 nm) polymeric films reported in the literature and obtained in this study, evaluated via different techniques.

Polymeric thin films can display colossal differences in their mechanical properties when compared to the bulk state.^79−81^ It was shown that the PDA in situ deposited coatings poses rather poor mechanical performance,^82^ and the techniques used to improve these properties such as thermal treatment process^82^ and blue diode laser annealing^83^ are unlikely to be applicable to PDA films from the air/water interface. For a versatile-functionalization platform, PDA free-standing films should be easily transferable on macro-scale surfaces and protect sensitive and easily degradable photoactive materials.^16^ Therefore, evaluating the mechanical property test results obtained for the PDA ultra-thin films to be transferred on the multifunctional substrates is important. To put these information in context, we have compared Young’s modulus values for various ultra-thin polymer films reported in the literature and this experiment (Figure 7). It can be seen that PDA free-standing films have a particularly high modulus of elasticity, especially DA/BA 1:3, when compared to available data for other similar materials.

The lack of error-bars for some materials in Figure 7 means that the measurement error was not given in the publication or its value was so small that the error-bar is smaller than the data point symbol. The comparison indicates that except PDA free-standing films, no polymeric nanometric-thin film exceeds the value of 10 GPa of Young’s modulus, including poly(methyl methacrylate) (PMMA) nanoscale thin films which are widely used in the emerging two-dimensional material nanotechnology applications.^84−86^ In particular, the outstanding properties of DA/BA 1:3 films should be emphasized here, which, within the measurement error, showed more than 2x higher Young’s modulus, with a similar thickness to PMMA.

For clarity, information presented in Figure 7 is summarized in Table 2 below. Importantly, the measurement techniques used were also specified.

**Table 2:** Summary of Young’s Modulus Measurements Data for Different Thin Polymer Films^a^

polymer thin film	thickness (nm)^b^	Young’s modulus (GPa)^b^	measurement method	refs
PMMA	40	7.8 ± 0.3	BLS	(87)
	19	3 ± 0.3	tensile tester	(88)
	5.8	9	AFM deflection test	(89)
	11.2	6		
	35	3.70		
poly(ethyl methacrylate) (PEMA)	9.8 ± 1.3	0.08 ± 0.03	surface wrinkling measurements	(90)
poly(*n*-propyl methacrylate) (PnPMA)	22.2 ± 2.7	0.039 ± 0.020		
poly(benzyl methacrylate) (PBzMA)	21.6 ± 1.5	0.042 ± 0.020		
poly(isobutyl methacrylate) (PibMA)	20.7 ± 1.9	0.05 ± 0.020		
polystyrene (PS)	30	2.8 ± 0.3	tensile tester	(91)
	40	5.7 ± 0.3	BLS	(87)
	100	5.6 ± 0.3		
polystyrene-*b*-polybutadiene-*b*-polystyrene block copolymer (SBS)	20	0.7	tensile tester	(92)
polyamide (PA)/cross-linked	200	1.50 ± 0.53	surface wrinkling measurements	(93)
PDA/genipin cross-linked	59.4 ± 4.0	7.9 ± 1.7	tensile tester	(94)
PDA	10 ± 1	12.5 ± 2.5	BLS	(76)
	13 ± 1	13.5 ± 2.5		
	**23.3 ± 3.0**	**14.4 ± 4.0**		**t.s**.
	55 ± 2	13 ± 4	nanoindentation	(14)
	**82.5 ± 9.9**	**14.1 ± 4.5**		**t.s**.
PDA/BA modified	**10.5 ± 1.5**	**18.3 ± 6.4**	BLS	**t.s.**
	**48.7 ± 6.1**	**24.1 ± 5.6**	nanoindentation	

a t.s.—this study.

b Error values are given as in the reference publications, without standardization.

**Figure 8:**
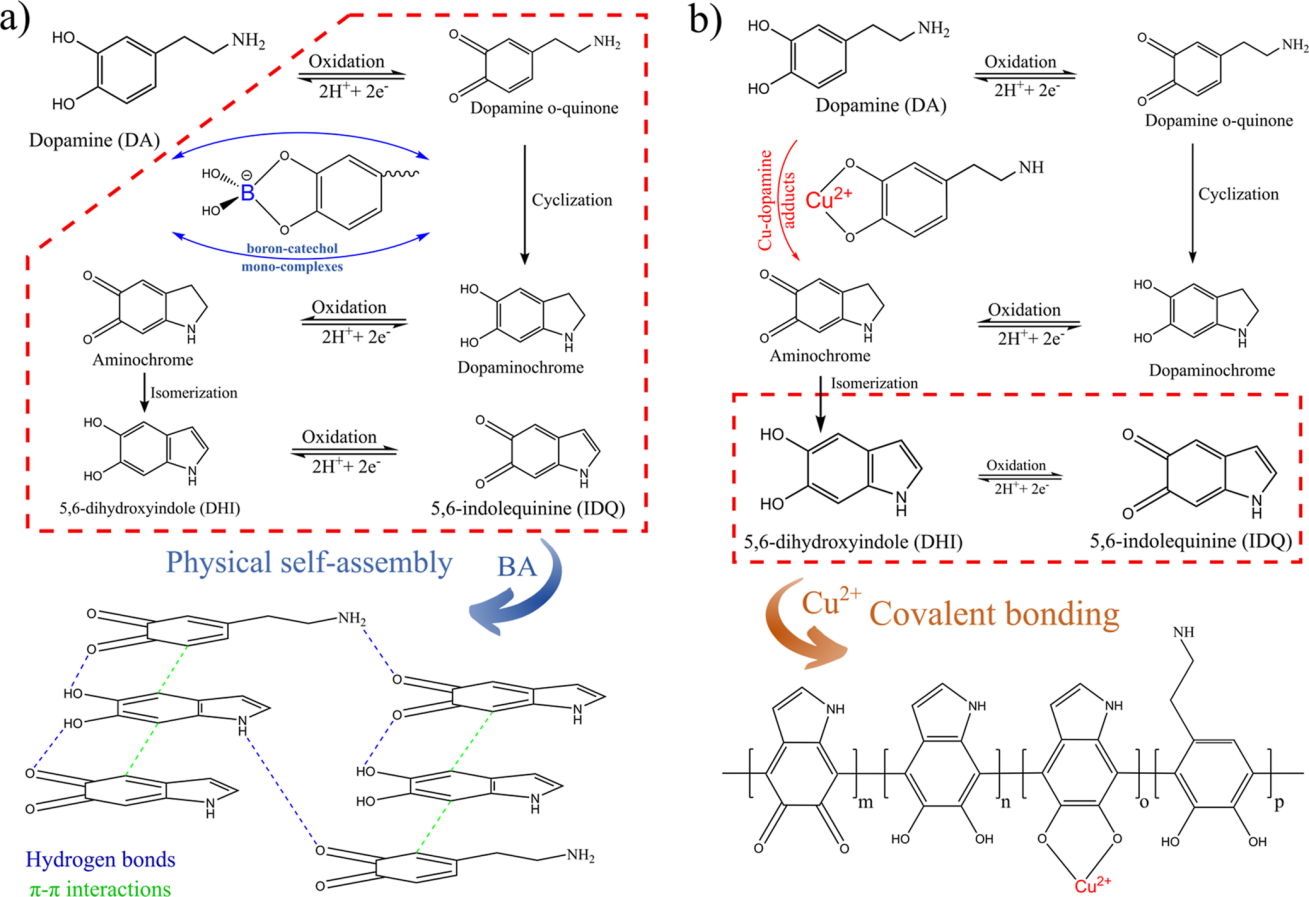
Scheme of the DA oxidation pathways with respect to used agents: (a) BA and (b) Cu^2+^ ions.

Finally, in Figure 8, we present a scheme of DA oxidation pathways with respect to used agents—BA (Figure 8a) and Cu^2+^ ions (Figure 8b). Although the final structure of the obtained materials results from various intermolecular interactions, the diagram is intended to detail the moderation properties of BA and Cu^2+^. The diagram shows the DA oxidation reaction, which proceeds according to a mechanism well-described in the literature,^6,52,95,96^ but the first step, that is the conversion of DA to *o*-quinone and subsequently to dopaminochrome, can be partially coursed by direct aminochrome synthesis through Cu–DA adducts or can be partially coursed and reversed by boron-catechol mono-complexes formation. Then, from the final products of the oxidation reaction, that is DHI and IDQ (and intermediate products in case of DA/BA films), PDA is formed as a result of different intermolecular processes, namely, covalent self-assembly and non-covalent self-assembly (π–π interactions and hydrogen bonds). Although very small amounts of metal ions (B and Cu) enter the final structure of PDA, their influence on the oxidation mechanism and cross-linking through covalent or coordination bonds is crucial.

## 4 Conclusions

In summary, we produced free-standing PDA films at the air/water interface with the use of two oxidation agents—BA and Cu^2+^ ions. We have effectively limited the arbitrary production of nanoparticles and aggregates of PDA nanoparticles suspended in a solution, especially for the films modified with BA. It is of great importance because these inclusions were previously reported to negatively affect the homogeneity and physico-chemical properties of the obtained films. Next, we showed, from the chemical point of view, the build from the nature-inspired polymer–PDA, but they show significant structural differences that is favored macromolecule aggregation type-through covalent (Cu^2+^) or non-covalent (BA) interactions, namely, π–π interactions and hydrogen bonds. Importantly for the oxidation mechanism, the conversion of DA to dopaminechrome through *o*-quinone can be partially coursed by direct aminochrome synthesis from Cu–DA adducts or can be partially coursed and reversed by boron-catechol mono-complex formation. Therefore, the agents used in the synthesis pose a vital role in the oxidation mechanism, with only small amounts of metals (B or Cu) detected in the final structure of PDA. XPS and FTIR revealed that catechol–Cu complex structural units are present in the Cu^2+^-modified films, while catechol-boron monocomplexes are unstable and are not embedded in the PDA structure. A here-only trace amount of boron was present in the free form. This allows for a better understanding of the self-assembly mechanism of molecules at the air/water interface and the formation of the thin free-standing films.

Furthermore, nanoindentation and BLS experiments revealed advantageous mechanical properties of BA-modified films, specifically its relatively large Young’s modulus and a finite residual stress. We postulate that they result from the favorably occurring non-covalent self-assembly mechanism. In combination with the results of our previous research, we have achieved exceptional control over the manufacturing process of these materials, that is, a precise measurement of their thickness growth in real-time, reduction of impurities, and selective self-assembly mechanism toward specific mechanical properties.

So far, the exploitation of PDA-thin films in applications such as photocatalytic enhancement and hierarchical architectures has been limited to in situ deposited PDA coatings, which are inherently mechanically poor and lack homogeneity and smoothness. Our study opens new perspectives for using mechanically resilient, free-standing films, with large-scale perspective, especially in applications in such as phononics, thermoelectrics, or nanometric-thin-layered composites for energy applications, which are applications where robust self-supporting functional PDA films might be needed. Additionally, due to the air/water nature of the films, large scalability can be achieved, reaching lateral sizes in the 10 s of centimeters, suitable for industrial photovoltaics or other energy applications.

Finally, our work provides unique insights into the mechanical (plastic and elastic) response of PDA films and the relationship between the structure, oxidation, and functionality of these films. It paves the way for future applications in layered nanocomposite engineering, where mechanically different but chemically compatible polymeric films can be integrated easily with other prospective materials, such as 2D van der Waals materials, resulting in the development of unique hybrid laminar architectures.

## Supporting Information Available

Scooping free-standing film-transferring technique; presentation of the detection limit in the new version of the SR setup; films from the air/water interface before and after transferring; UV–vis full spectra of the reaction solutions; Petri dishes after 24 h of oxidation and removal of the reaction mixture; ζ of the nanoparticles in the solution during the DA oxidation; AFM topography images of the films after 72 h of oxidation; roughness rms for a free-standing films after transferring on Si substrates; XPS data for the chemical analysis of the DA film; XPS full spectra and high-resolution spectra of the DA film; XPS data for the chemical analysis of the DA/BA 1:3 film; XPS full spectra and high-resolution spectra of the DA/BA 1:3 film; XPS data for the chemical analysis of the DA/Cu 1:3 film; XPS full spectra and high-resolution spectra of the DA/Cu 1:3 film; quantitative analysis of the XPS sp^2^/sp^3^ carbon subpeak areas; EDX chemical analysis of the DA/Cu 1:3 sample; results of the EDX chemical analysis of the DA/Cu 1:3 sample; deconvoluted part of the FTIR spectra in range 2800–3700 cm^–1^; Raman spectra; 2D (*G*′) peak intensity and maximum position comparison; X-ray diffractogram of the DA, DA/BA 1:3, and DA/Cu 1:3 films; peaks and structural parameters obtained from the XRD diffractogram; AFM topography of the DA sample after 12 h of oxidation; and AFM topography of the DA/BA 1:3 sample after 12 h of oxidation (PDF)

## Notes

The raw/processed data required to reproduce these findings cannot be shared at this time as the data also forms part of an ongoing study. Data might be shared upon reasonable request to the corresponding author.
