# Supplementary material for: Control of Intermolecular Interactions toward the Production of Free-Standing Interfacial Polydopamine Films
Source: ACS Appl Mater Interfaces. 2023 Jul 25;15(30):36922–35. doi: 10.1021/acsami.3c05236 (PMC10401576; doi:10.1021/acsami.3c05236)
Supplement: Supplementary file 1 — am3c05236_si_001.pdf [file am3c05236_si_001.pdf]

Supporting Information

Control of Intermolecular Interactions Towards the Production of Free-standing Interfacial Polydopamine Films

Jakub Szewczyk*[1,2], Visnja Babacic[3], Adam Krysztofik[4], Olena Ivashchenko[1], Mikołaj Pochylski[4], Robert Pietrzak[3], Jacek Gapiński[4], Bartłomiej Graczykowski[4], Mikhael Bechelany[2,5], Emerson Coy[1]*

1 NanoBioMedical Centre, Adam Mickiewicz University, Wszechnicy Piastowskiej 3, 61-614, Poznan, Poland.

2 Institut Européen des Membranes, IEM, UMR 5635, Univ Montpellier, CNRS, ENSCM Place Eugène Bataillon, 34095 Montpellier cedex 5, France

3 Faculty of Chemistry, Adam Mickiewicz University, Uniwersytetu Poznańskiego 8, 61-614 Poznań, Poland

4 Faculty of Physics, Adam Mickiewicz University, ul. Uniwersytet Poznańskiego 2, 61-614 Poznań, Poland.

5 Gulf University for Science and Technology, GUST, Kuwait

*** Corresponding authors:** jaksze3@amu.edu.pl, coyeme@amu.edu.pl

Figure S1	Scheme of the scooping free-standing films transferring technique……………	S2
Figure S2	Presentation of the detection limit in the new version of the SR set-up ………..	S3
Figure S3	films from the air/water interface before and after transferring ………………..	S4
Figure S4	UV-Vis full spectra of the reaction solutions ……………………………......	S5
Figure S5	Petri dishes after 24 hours of oxidation and removal of the reaction mixture ….	S6
Figure S6	ζ of the of the nanoparticles in the solution during the dopamine oxidation …...	S7
Figure S7	AFM topography images of the films after 72h oxidation……………………...	S8
Table S1	Roughness RMS for a free-standing films after transferring on Si substrates….	S8
Table S2	XPS data for the chemical analysis of the DA film …………………………….	S9
Figure S8	XPS full spectra and high-resolution spectra of the DA film …………………...	S10
Table S3	XPS data for the chemical analysis of the DA:BA 1:3 film …………………….	S11
Figure S9	XPS full spectra and high-resolution spectra of the DA:BA 1:3 film ………….	S12
Table S4	XPS data for the chemical analysis of the DA:Cu 1:3 film …………………….	S13
Figure S10	XPS full spectra and high-resolution spectra of the DA:Cu 1:3 film …………..	S14
Table S5	Quantitative analysis of the XPS sp2/sp3 Carbon subpeaks areas………………	S15
Figure S11	EDX chemical analysis of the DA:Cu 1:3 sample ……………………………...	S16
Table S6	Results of the EDX chemical analysis of the DA:Cu 1:3 sample……………….	S16
Figure S12	Deconvoluted part of the FTIR spectra in range 2800-3700 cm^{-1} ………………	S17
Figure S13	Raman spectra, 2D (G’) peak intensity and maximum position comparison.......	S17
Figure S14	X-ray diffractogram of the DA, DA:BA 1:3 and DA:Cu 1:3 films……………..	S18
Table S7	Peaks and structural parameters obtained from the XRD diffractogram..………	S18
Figure S15	AFM topography of the DA sample after 12h of oxidation…………………….	S19
Figure S16	AFM topography of the DA:BA 1:3 sample after 12h of oxidation……………	S20

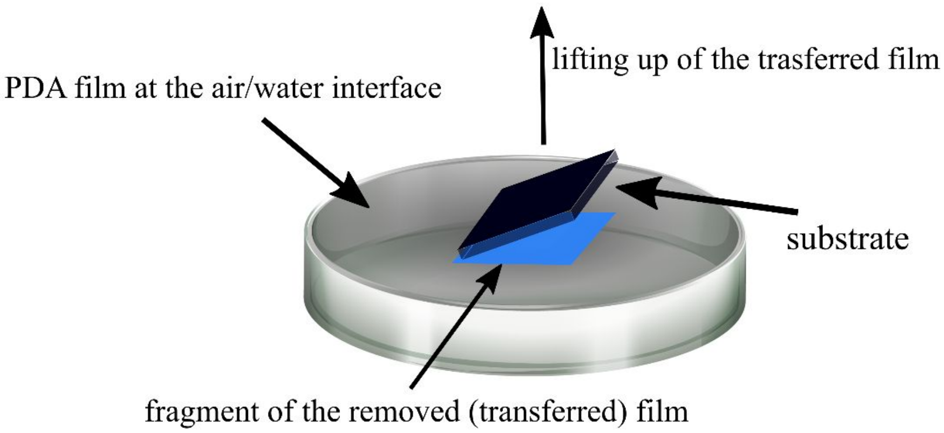

Figure S1. Scheme of the scooping transferring technique

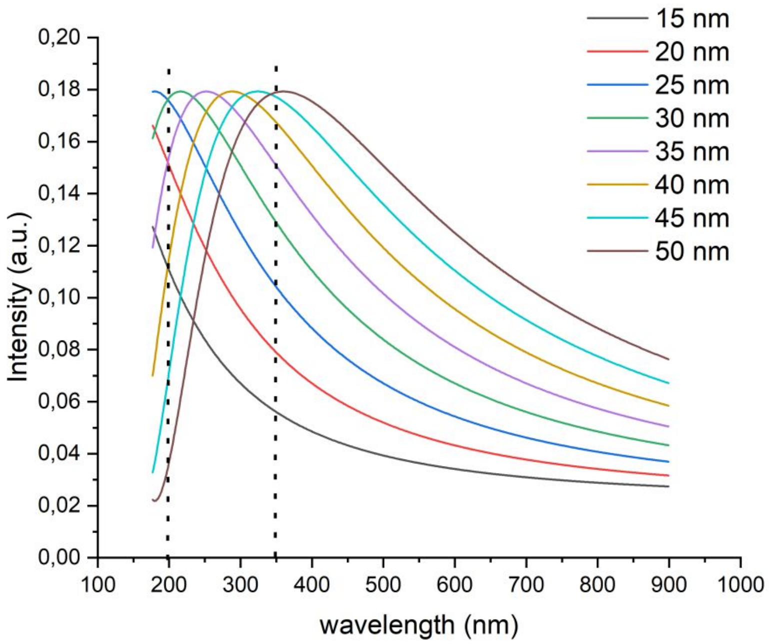

Figure S2. Presentation of the detection limit in the new version of the SR set-up.

The principle of the operation of this set-up was well described in our previous publication[1]

The relative intensity of light reflected from the whole film is given by Equation:

$$\frac{I_{refl}}{I_0} = R_1 + R_2T_1^2 \pm 2T_1\sqrt{R_1R_2}cos\left(2\pi\frac{2nd}{\lambda}\right)$$

Where:

- d - film thickness
- λ - light wavelength
- $R_{1,2}$ - reflection coefficients from upper and lower surfaces of the PDA films, respectively. General formula is as follows:

  $$R = \left(\frac{n_1 - n_2}{n_1 + n_2}\right)^2$$

  Where:

  n_1 and n_2 - the refractive indices of media 1 and 2

  Taking $n_{water} = 1.33$ [2], $n_0 = 1.0$ and $n_{PDA} = 1.80$ [3], we obtain $R_1 = 0.0816$ and $R_2 = 0.0225$
- $T_1 = 1 - R_1$

The dashed line shows the range of analyzed wavelengths. Colored graphs correspond to the the relative intensity of light reflected from the whole film function. The position of the first maximum of the function finds the thickness of the film.

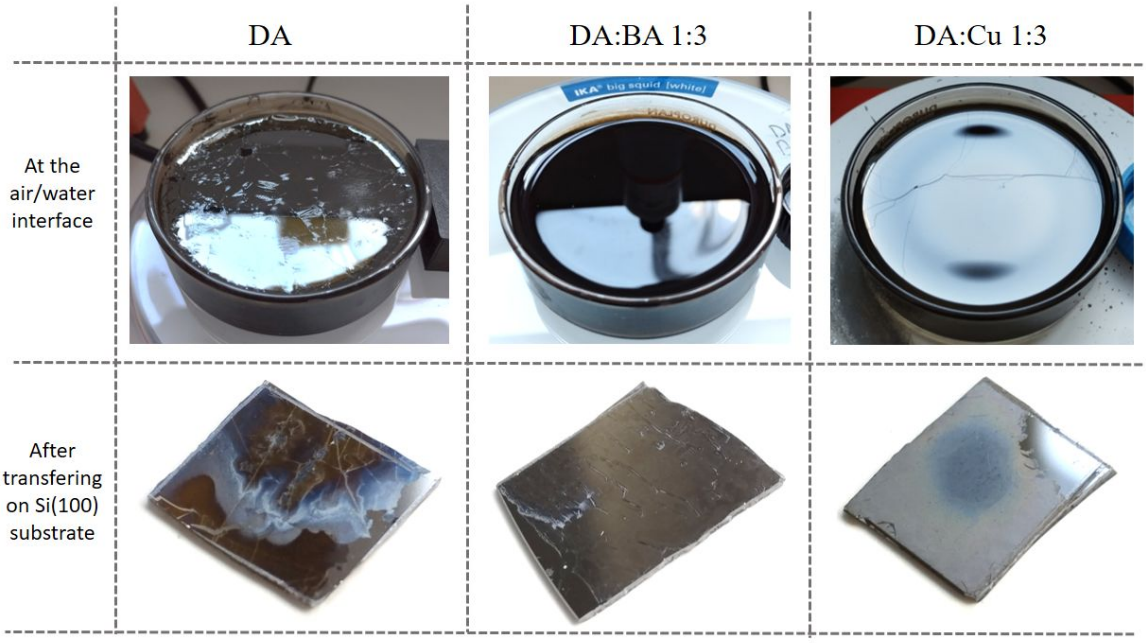

Figure S3: Photographs of the DA, DA:BA 1:3 and DA:Cu 1:3 films from the air/water interface before and after transferring on the Si substrates. The choice of just these 3 groups is not arbitrary and results from the experiences that are discussed later in the manuscript.

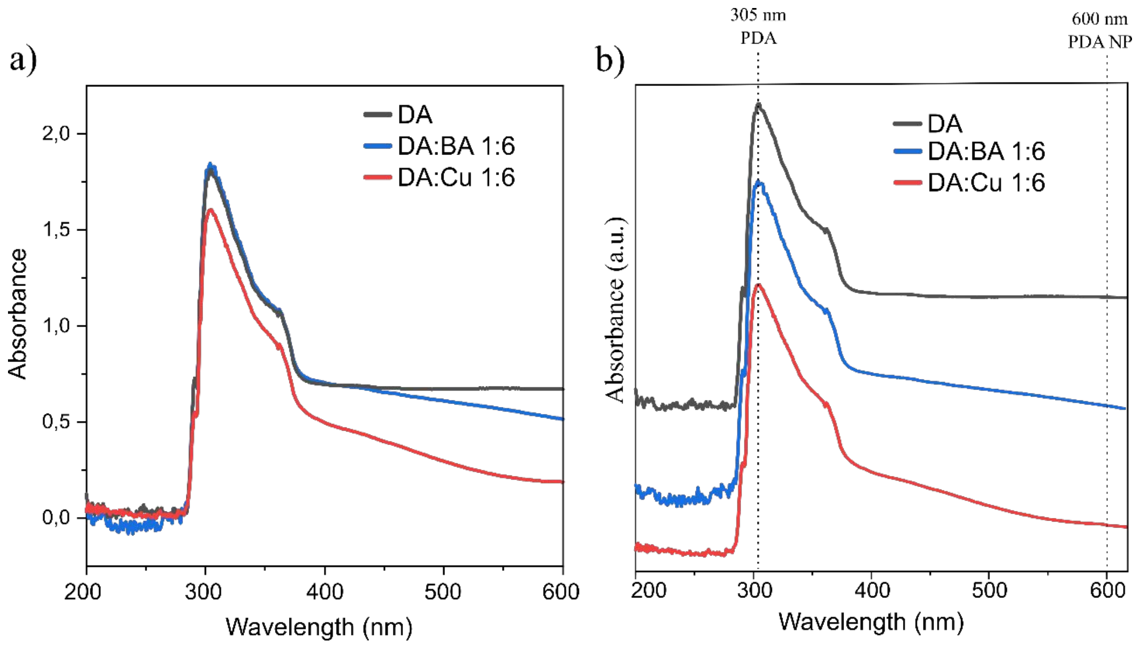

Figure S4. UV-Vis full spectra of the reaction solutions a) keeping the actual absorbance units on the y-axis, b) in arbitrary units (to stretch graphs).

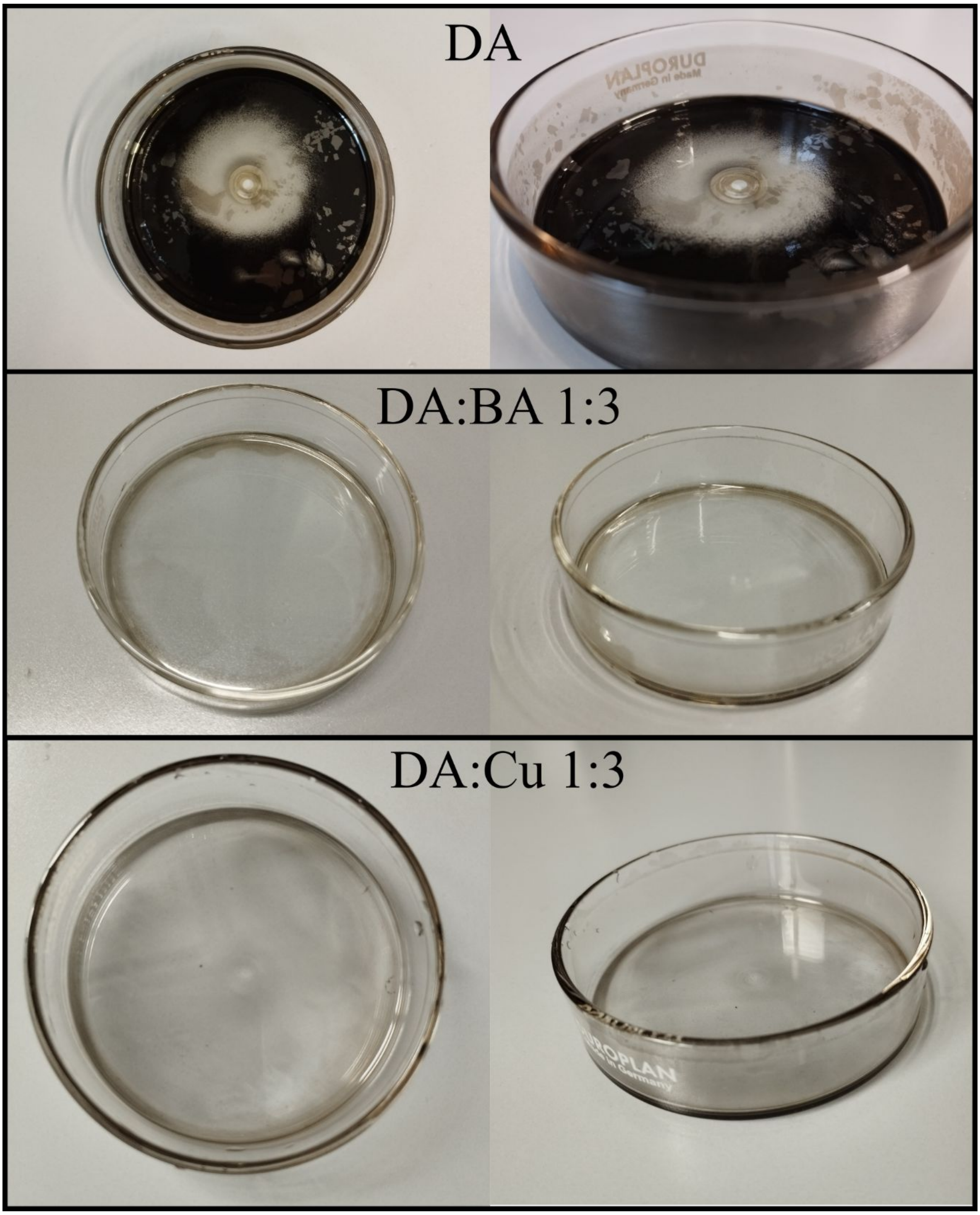

Figure S5. Petri dishes after 24 hours of oxidation and removal of the reaction mixture. Sedimentation of the PDA in form of large nanoparticle and aggregates at the bottom and walls of the Petri dish.

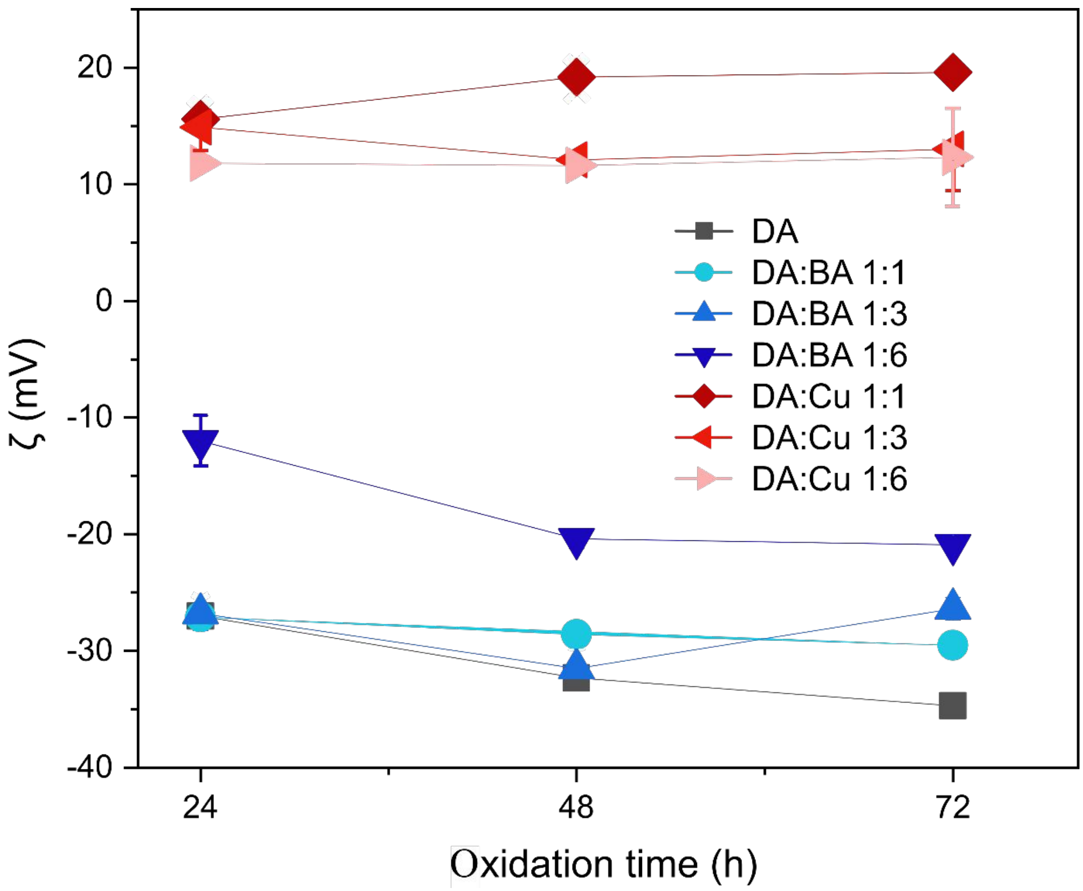

Figure S6: ζ (zeta potential) of the of the PDA nanoparticles in the solution during 72 h of the dopamine oxidation modified with BA and Cu^{2+}

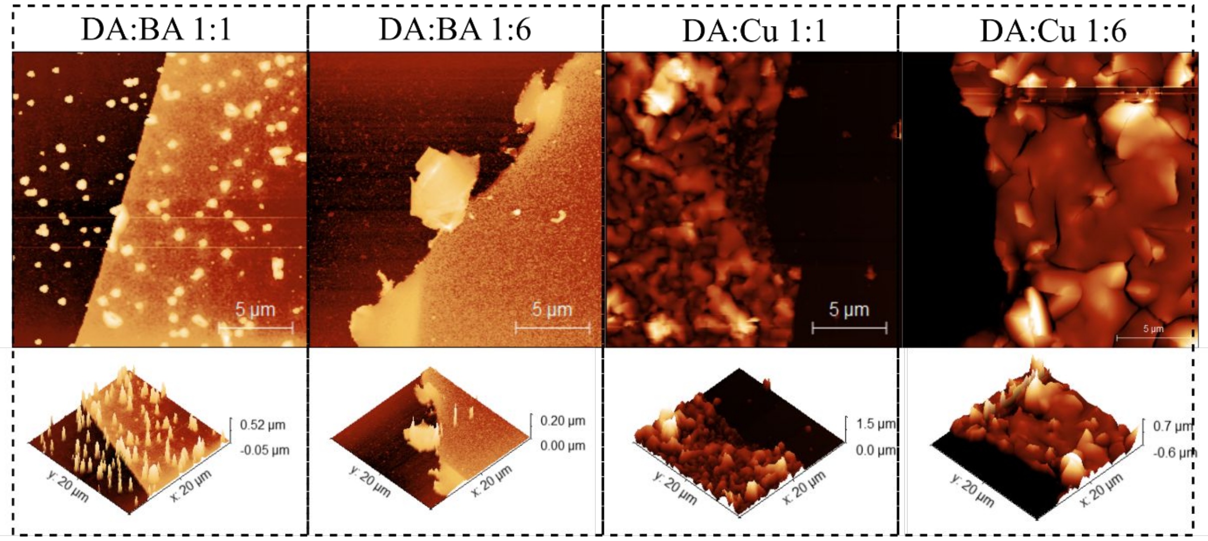

Figure S7: AFM topography images of the films after 72h oxidation at the air/water interface of the DA:BA 1:1, DA:BA 1:6, DA:Cu 1:1 and DA:Cu 1:6

Table S1: AFM roughness RMS results for free-standing films after transferring on silicon substrates

Sample	Roughness RMS (nm)	Std. Dev. (nm)
DA	24.89	15.72
DA:BA 1:1	18.42	13.23
DA:BA 1:3	13.17	1.89
DA:BA 1:6	2.26	1.01
DA:Cu 1:1	48.19	13.89
DA:Cu 1:3	49.94	15.72
DA:Cu 1:6	50.76	17.49

Table S2. XPS data for the chemical analysis of the DA film

**Region**	**Atomic percent (%) (+/- 0.01)**			
O 1s	12.37			
C 1s	78.04			
N 1s	5.15			
Si 2p	3.50			
Cl 2p	0.94			
**High Resolution Spectra**				
**Binding energy**	**spin splitting**	**Assignment**	**FWHM**	**Area (%) (+/- 0.01)**
**C 1s**				
284.39	-	sp2 (C=C)	0.97	9.99
284.80	-	sp3 (C-C, C-H)	1.52	38.38
286.15	-	C-O, C-N	1.52	35.76
287.55	-	C=O	1.52	9.26
288.76	-	O-C=O	1.52	3.25
290.73	-	pi-pi	3.00	3.36
**O 1s**				
531.49	-	(C=O)	2.16	23.66
532.88	-	(C-O, SiO_2)	2.16	76.34
**Si 2p**				
99.35	2p 3/2	metal Si	0.69	18.60
99.96	2p ½	metal Si	0.70	18.24
101.97	/	Si-O	1.35	61.19
103.23	/	SiO2	1.35	16.18
**N 1s**				
398.54	-	=N-C	1.65	7.18
400.08	-	C-N-C (N-C=O)	1.65	75.14
401.88	-	N-C, N-O	1.65	17.68
**Cl 2p**				
Cl in small concentration				

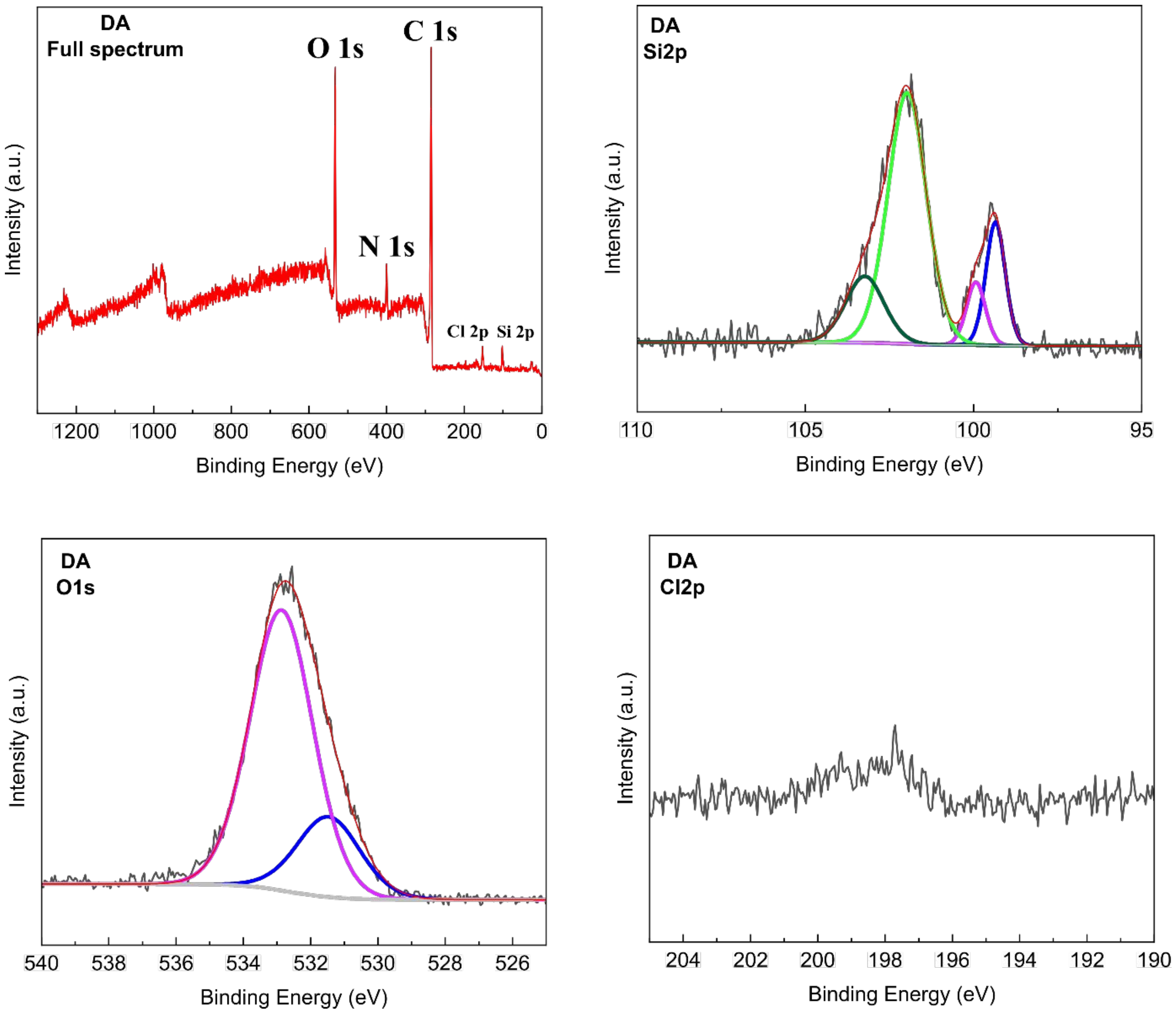

Figure S8. X-Ray photoelectron full spectra and high-resolution spectra of the Si2p, O1s, Cl2p regions for the DA film.

Table S3. XPS data for the chemical analysis of the DA:BA 1:3 film

Region	Atomic percent (%) (+/- 0.01)			
O 1s	22.59			
C 1s	61.92			
N 1s	8.29			
Si 2p	3.93			
Cl 2p	1.89			
B 1s	1.38			
**High Resolution Spectra**				
**Binding energy**	**spin splitting**	**Assignment**	**FWHM**	**Area (%) (+/- 0.01)**
**C 1s**				
284.32	-	sp2 (C=C)	0.78	4.77
284.80	-	sp3 (C-C, C-H)	1.42	47.96
286.32	-	C-O, C-N, C-S	1.42	35.04
287.95	-	C=O	1.42	5.72
289.07	-	O-C=O	1.42	2.84
292.09	-	pi-pi	3.50	3.67
**O 1s**				
532.18	-	C=O	2.1	58.36
532.85	-	C-O, SiO_2	2.1	41.64
**Si 2p**				
99.87	2p 3/2	metal Si	1.2	7.27
100.50	2p 1/2	metal Si	0.7	7.13
102.11	/	Si-O	1.44	80.21
104.28	/	SiO2	1.44	5.39
**N 1s**				
398.32	-	=N-C	1.64	3.76
400.04	-	C-N-C (N-C=O)	1.64	55.35
401.73	-	N-C, N-O	1.64	40.89
**Cl 2p + B 1s**				
197.88	2p 3/2	Cl	1.26	40.10
199.45	2p 1/2	Cl	1.21	39.10
192.06	/	B (B2O3)	1.31	20.80

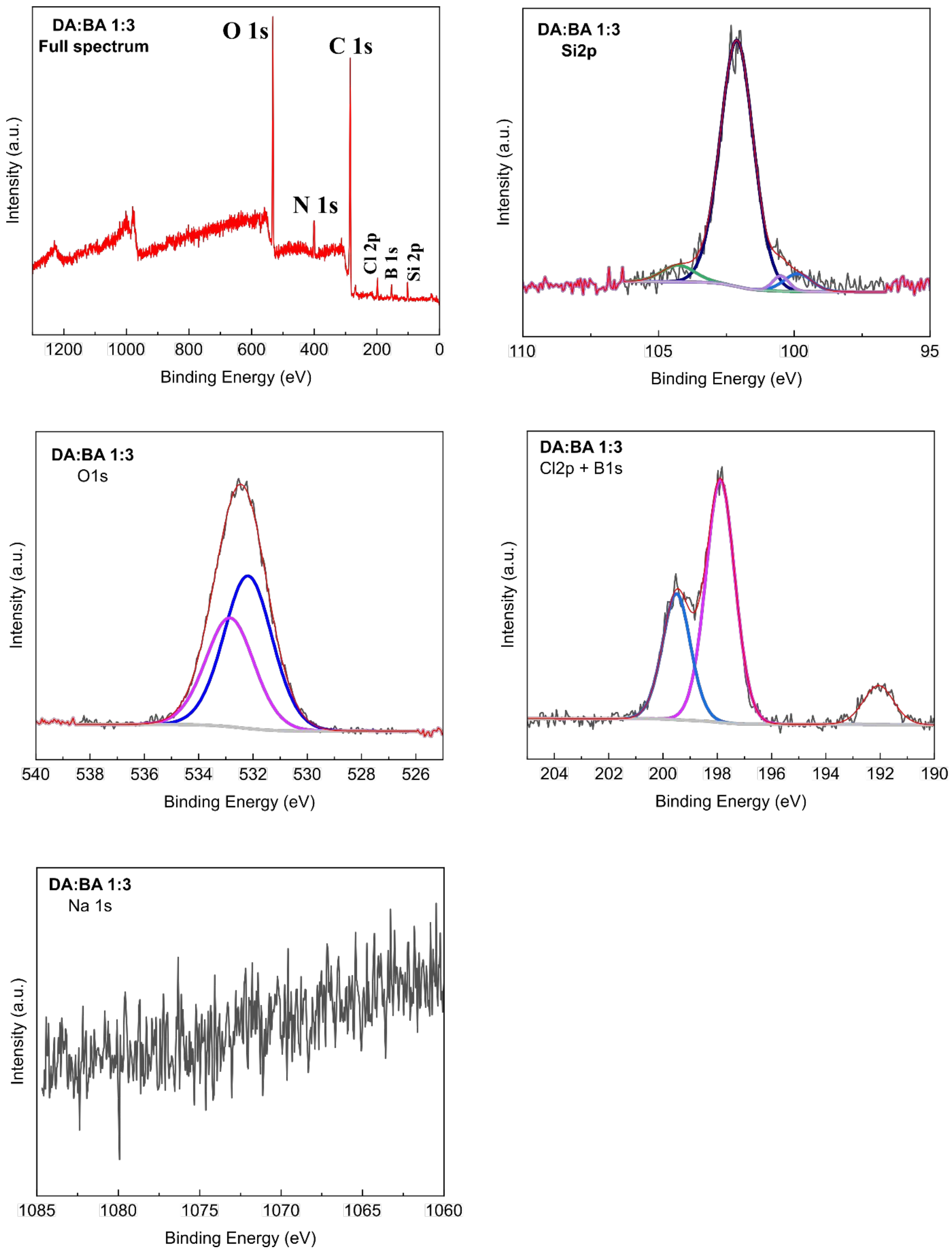

Figure S9. X-Ray photoelectron full spectra and high-resolution spectra of the Si2p, O1s, Cl2p, B1s regions for the DA:BA 1:3 film.

Table S4. XPS data for the chemical analysis of the DA:Cu 1:3 film.

Region	Atomic percent (%) (+/- 0.01)			
O 1s	22.07			
C 1s	50.25			
N 1s	6.47			
Si 2p	14.95			
Cu 1s	1.85			
S 2p	4.41			
**High Resolution Spectra**				
**Binding energy**	**spin splitting**	**Assignment**	**FWHM**	**Area (%) (+/- 0.01)**
**C 1s**				
284.30	-	sp2 (C=C)	1.26	29.89
284.80	-	sp3 (C-C, C-H)	1.57	20.60
285.69	-	C-O, C-N, C-S	2.00	36.56
287.92	-	C=O	2.22	12.96
**O 1s**				
531.29	-	C=O, CuO	1.84	49.91
532.65	-	C-O, SiO_2	1.84	50.09
**Si 2p**				
99.21	2p 3/2	metal Si	0.71	40.09
99.83	2p 1/2	metal Si	0.71	39.30
101.64	/	Si-O	1.58	9.94
103.34	/	SiO2	1.58	10.66
**N 1s**				
398.28	-	=N-C	1.94	8.80
399.79	-	C-N-C (N-C=O)	1.94	76.17
401.69	-	N-C, N-O	1.94	15.03
**S 2p + Si 1s**				
164.62	2p 3/2	S	1.77	11.43
165.80	2p 1/2	S	1.38	11.20
168.15	/	S	2.72	68.64
170.94	/	S	2.22	8.73
**Cu 2p**				
934.46	2p 3/2	CuO	2.88	34.16
954.44	2p 1/2	CuO	3.71	33.90
939.38	/	Satelite	3.87	8.88
943.73	/	Satelite	5	14.92
962.39	/	satelite	4.61	8.15

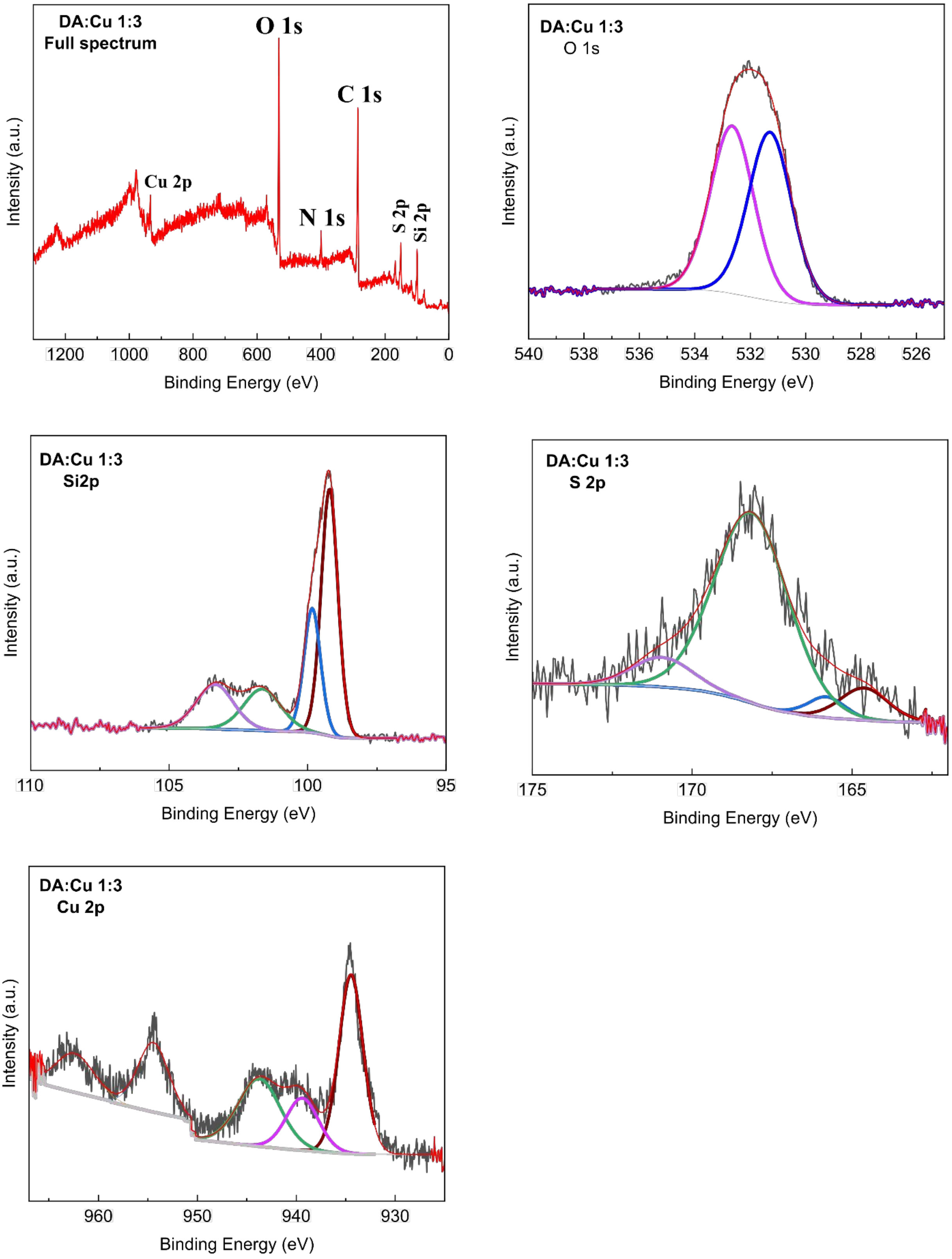

Figure S10. X-Ray photoelectron full spectra and high-resolution spectra of the Cu2p, O1s, S2p, Si2p regions for the DA:Cu 1:3 film.

Table S5. Quantitative analysis of the XPS sp2/sp3 Carbon subpeaks areas.

	**DA**	**DA:BA**	**DA:Cu**
sp2 Carbon subpeak area (%)	9.99	4.77	29.89
sp3 Carbon subpeak area (%)	38.38	47.96	20.60
sp2/sp3 Carbon ratio	**0.26**	**0.10**	**1.45**

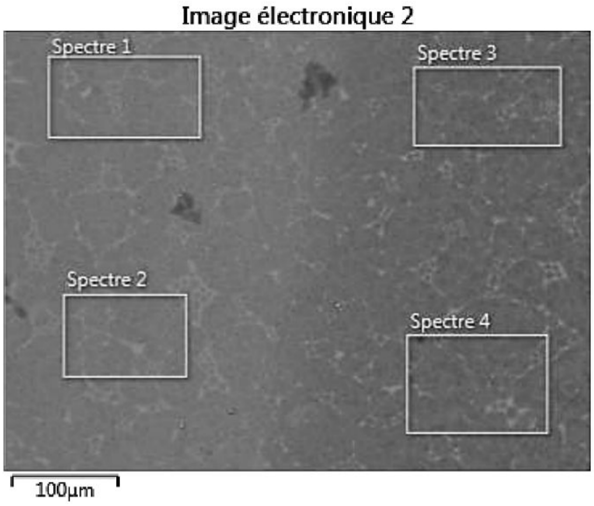

Figure S11. EDX chemical analysis of the DA:Cu 1:3 sample.

Table S6. Results of the EDX chemical analysis of the DA:Cu 1:3 sample.

Spectrum	Element	% Mass (+/- 0.01)	% Atomic (+/- 0.01)
1	C	35.56	53.29
	N	4.06	5.22
	O	6.89	7.75
	Si	51.49	33.00
	S	0.16	0.09
	Cl	0.57	0.29
	Cu	1.28	0.36
	Total:	100.00	100.00
2	C	35.44	53.31
	N	3.70	4.78
	O	6.86	7.75
	Si	51.91	33.40
	S	0.11	0.06
	Cl	0.59	0.30
	Cu	1.38	0.39
	Total:	100.00	100.00
3	C	37.60	54.44
	N	4.68	5.82
	O	10.20	11.08
	Si	44.24	27.40
	S	0.22	0.12
	Cl	1.42	0.70
	Cu	1.63	0.45
	Total:	100.00	100.00
4	C	37.62	54.46
	N	4.52	5.61
	O	10.43	11.33
	Si	44.10	27.30
	S	0.25	0.13
	Cl	1.47	0.72
	Cu	1.62	0.44
	Total:	100.00	100.00

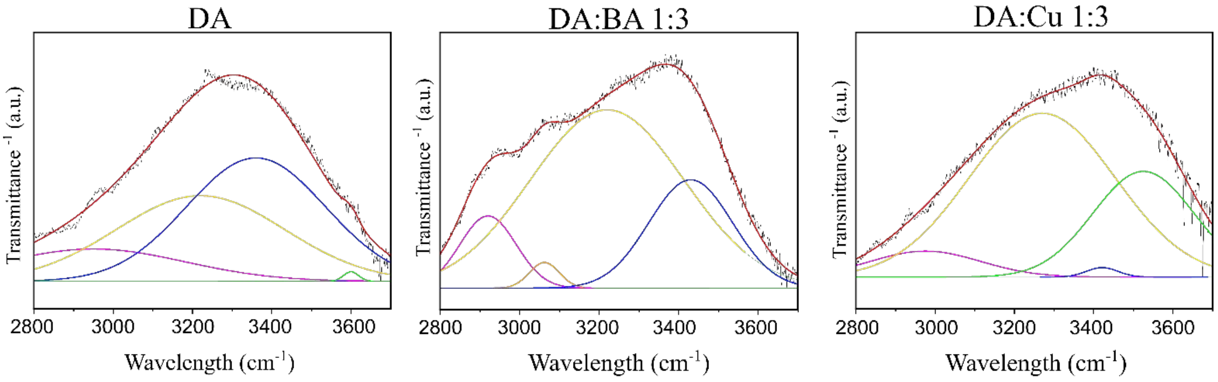

	center (cm-1)	int. area (%)
	2950	12.5
	–	–
	3220	39.6
	3360	47.5
	3600	0.4

	center (cm-1)	int. area (%)
	2920	9.6
	3062	1.9
	3220	66.3
	3430	22.2
	–	–

	center (cm-1)	int. area (%)
	2975	7.1
	–	–
	3269	65.2
	3421	0.8
	3525	26.9

Figure S12. Deconvoluted part of the FTIR spectra in range 2800-3700 cm^{-1}.

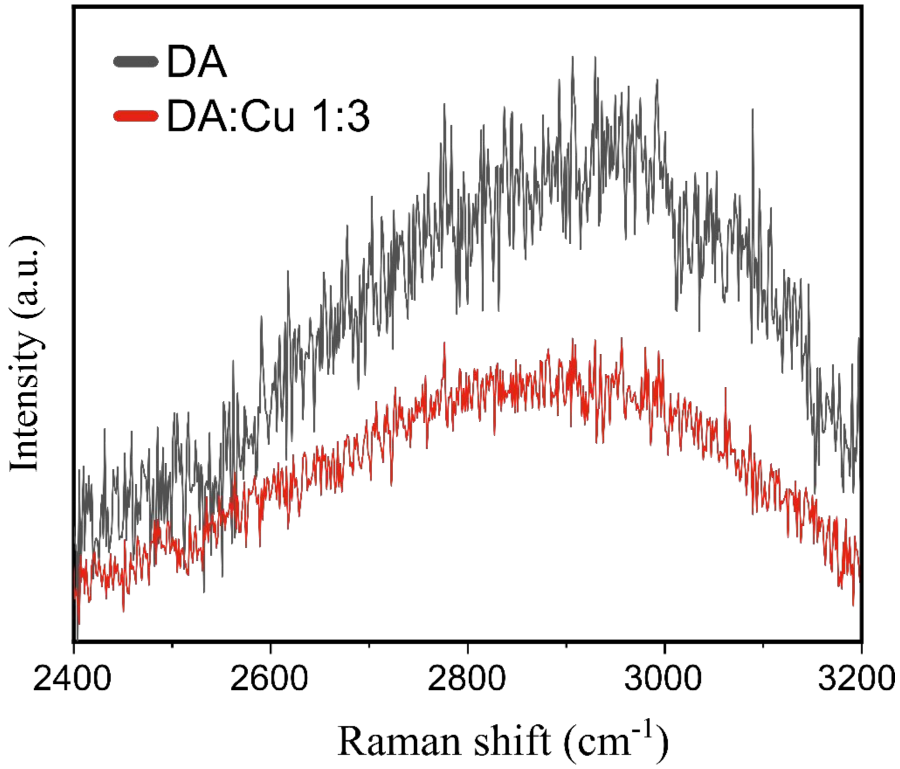

Figure S13. Raman spectra, 2D (G') peak intensity and maximum position comparison for DA and DA:Cu 1:3 films.

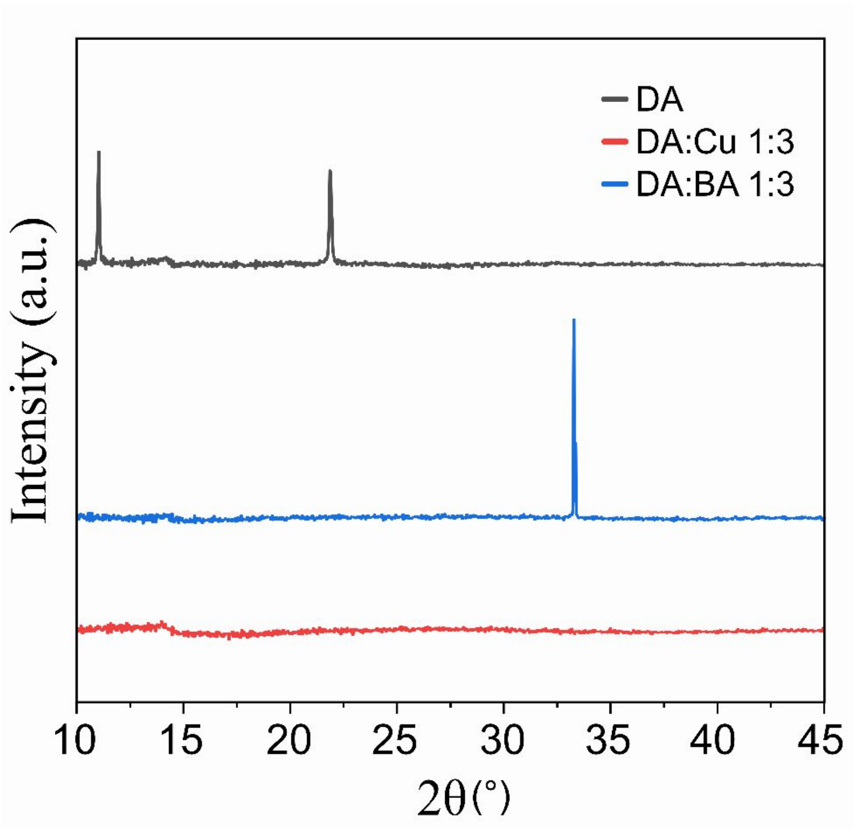

Figure S14. X-ray diffractogram of the DA, DA:BA 1:3 and DA:Cu 1:3 films.

XRD Peaks have been signed and characterized in the **Table S5**.

Table S7. Peaks and structural parameters obtained from the XRD diffractogram.

Sample	2θ (°)	FWHM (°)	d (Å) (+/- 0.01)	L avg. (nm) (+/- 0.01)
DA	11.028	0.09897	8.01	1.24
	21.883	0.14613	4.06	
DA:BA 1:3	33.211	0.03373	2.69	4.14

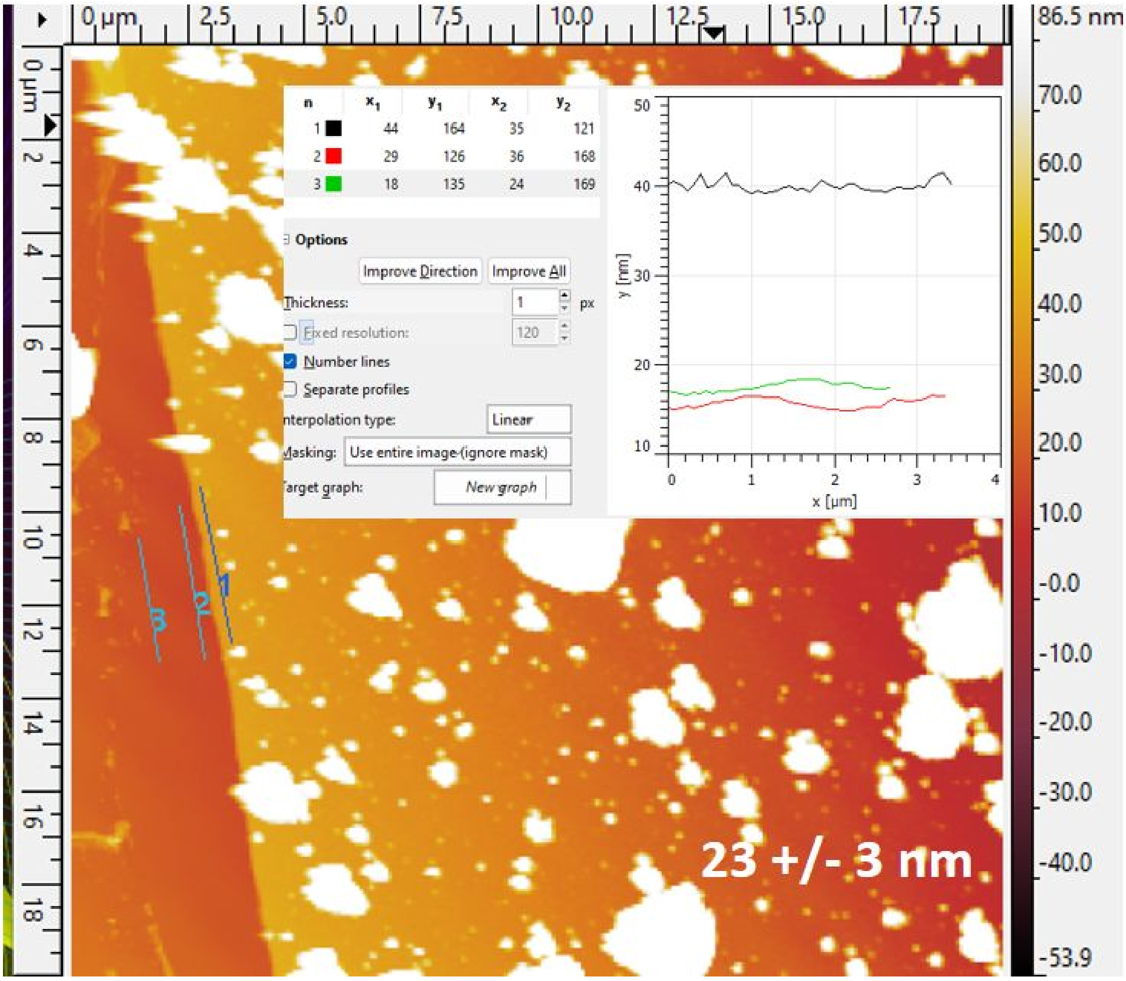

Figure S15. AFM topography of the DA sample after 12h of oxidation.

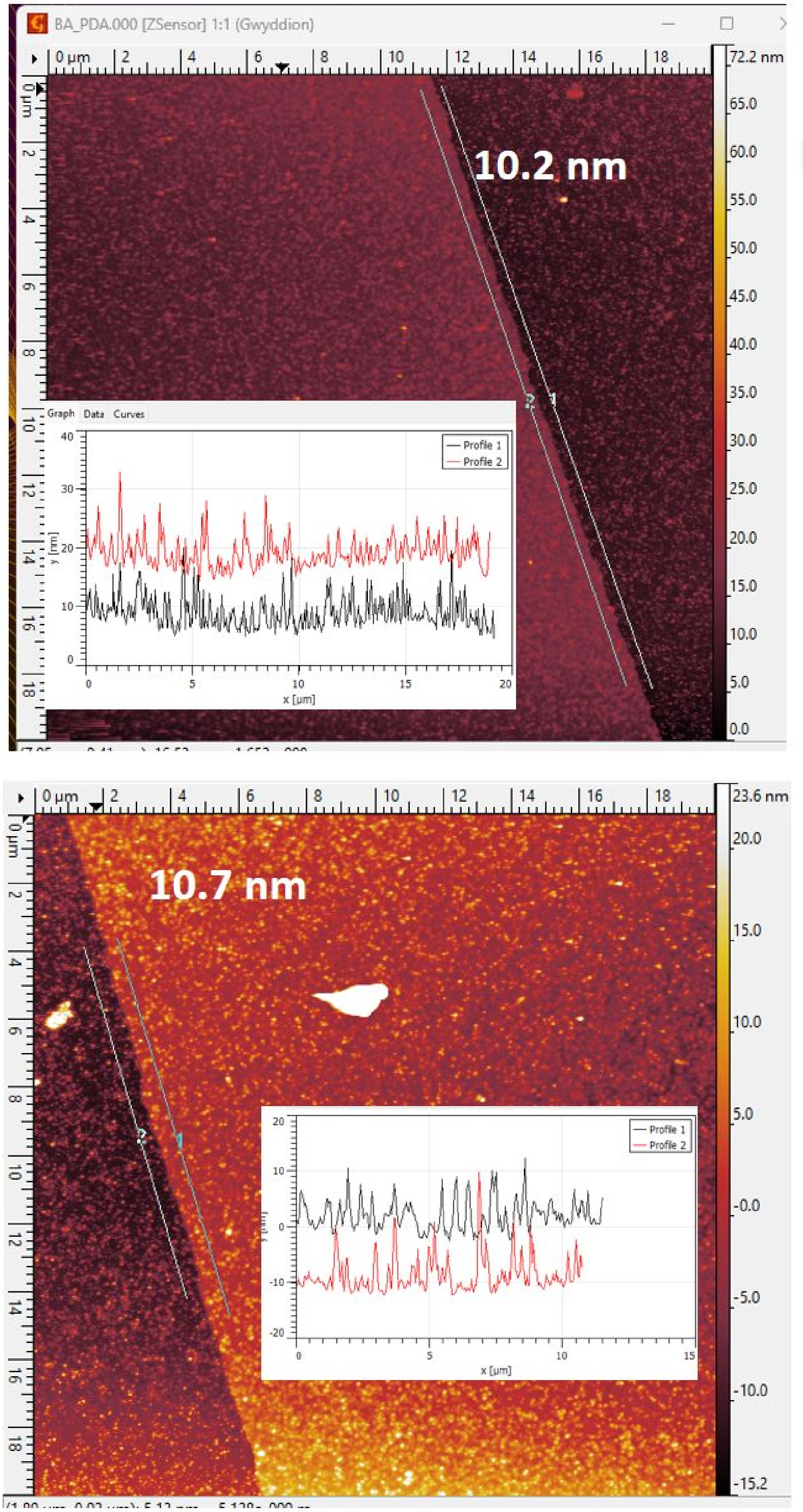

Figure S16. AFM topography of the DA:BA 1:3 sample after 12h of oxidation.

References

(1) Szewczyk, J.; Pochylski, M.; Szutkowski, K.; Kempiński, M.; Mrówczyński, R.; Iatsunskyi, I.; Gapiński, J.; Coy, E. In-Situ Thickness Control of Centimetre-Scale 2D-Like Polydopamine Films with Large Scalability. *Mater. Today Chem.* **2022**, *24*, 100935. https://doi.org/https://doi.org/10.1016/j.mtchem.2022.100935.

(2) Thormählen, I.; Straub, J.; Grigull, U. Refractive Index of Water and Its Dependence on Wavelength, Temperature, and Density. *J. Phys. Chem. Ref. Data* **1985**, *14* (4), 933–945. https://doi.org/10.1063/1.555743.

(3) Qie, R.; Zajforoushan Moghaddam, S.; Thormann, E. Parameterization of the Optical Constants of Polydopamine Films for Spectroscopic Ellipsometry Studies. *Phys. Chem. Chem. Phys.* **2021**, *23* (9), 5516–5526. https://doi.org/10.1039/d0cp04796a.
